# Andrographolide Promotes Interaction Between Endothelin-Dependent EDNRA/EDNRB and Myocardin-SRF to Regulate Pathological Vascular Remodeling

**DOI:** 10.3389/fcvm.2021.783872

**Published:** 2022-01-20

**Authors:** Wangming Hu, Xiao Wu, Zhong Jin, Zheng Wang, Qiru Guo, Zixian Chen, Song Zhu, Haidi Zhang, Jian Huo, Lingling Zhang, Xin Zhou, Lan Yang, Huan Xu, Liangqing Shi, Yong Wang

**Affiliations:** ^1^College of Basic Medicine, Chengdu University of Traditional Chinese Medicine, Chengdu, China; ^2^School of Ethnic Medicine, Chengdu University of Traditional Chinese Medicine, Chengdu, China; ^3^Hospital of Chengdu University of Traditional Chinese Medicine, Chengdu University of Traditional Chinese Medicine, Chengdu, China; ^4^Chengdu Women's and Children's Central Hospital, School of Medicine, University of Electronic Science and Technology of China, Chengdu, China

**Keywords:** Andrographolide, EDNRA, EDNRB, Myocardin-SRF, CArG box, pathological vascular remodeling

## Abstract

**Introduction:**

Pathological vascular remodeling is a hallmark of various vascular diseases. Smooth muscle cell (SMC) phenotypic switching plays a pivotal role during pathological vascular remodeling. The mechanism of how to regulate SMC phenotypic switching still needs to be defined. This study aims to investigate the effect of Andrographolide, a key principle isolated from Andrographis paniculate, on pathological vascular remodeling and its underlying mechanism.

**Methods:**

A C57/BL6 mouse left carotid artery complete ligation model and rat SMCs were used to determine whether Andrographolide is critical in regulating SMC phenotypic switching. Quantitative real-time PCR, a CCK8 cell proliferation assay, BRDU incorporation assay, Boyden chamber migration assay, and spheroid sprouting assay were performed to evaluate whether Andrographolide suppresses SMC proliferation and migration. Immunohistochemistry staining, immunofluorescence staining, and protein co-immunoprecipitation were used to observe the interaction between EDNRA, EDNRB, and Myocardin-SRF.

**Results:**

Andrographolide inhibits neointimal hyperplasia in the left carotid artery complete ligation model. Andrographolide regulates SMC phenotypic switching characterized by suppressing proliferation and migration. Andrographolide activates the endothelin signaling pathway exhibited by dramatically inducing EDNRA and EDNRB expression. The interaction between EDNRA/EDNRB and Myocardin-SRF resulted in promoting SMC differentiation marker gene expression.

**Conclusion:**

Andrographolide plays a critical role in regulating pathological vascular remodeling.

## Introduction

Pathological vascular remodeling exhibits smooth muscle cell (SMC) phenotypic switching (1). SMCs have remarkable plasticity, and can undergo phenotypic switching in response to vascular endothelium damage (2), altered blood flow (3), inflammatory stimulation (4), or various disease conditions. Typical SMC phenotypic switching from the differentiation stage to the dedifferentiation stage is characterized by enhanced proliferation and decreased expression of SMC contractile-specific marker genes, including smooth muscle (SM) α-actin, smooth muscle myosin heavy chains (SM MHCs), smooth muscle myosin light chains, h1-calponin, and smooth muscle α-tropomyosin (1). SMC phenotypic switching is critical in initiation of vascular diseases, including atherosclerosis, post angioplasty restenosis, aneurysm, pulmonary hypertension, diabetic-related retinal vasculopathy, allograft vasculopathy, and transplantation associated vasculopathy (5, 6).

SMCs express differentiated marker genes. Tremendous progress has been made in serum response factor (SRF) homodimers with conserved CArG boxes and promoter-enhancer regions of several SMC differentiated marker genes to regulate SMC differentiated marker gene expression (1, 7, 8). The CArG box-dependent SMC differentiation marker genes include smooth muscle a-actin, Calponin, SM22a, and MHC. Whereas CArG box-independent SMC differentiation marker genes include FRNK, Smoothelin, and a1-integrin (1). Myocardin dramatically promotes the interaction between SRF and CArG boxes (9). Additional coactivators have been reported to enhance Myocardin and SRF-CArG boxes interaction, such as Prx1, stat3, Klf4, GATA4, Nkx3.2, and MRTFA/B (10–13).

The endothelin/sarafotoxin family comprises three isoforms, including ET-1, ET-2, and ET-3, which comprise similar structures with 21 amino acids (14, 15). Endothelins are produced primarily in the vascular endothelium, and the most potent vasoconstrictors through activation of two G protein-coupled receptors are endothelinA (EDNRA) and endothelinB (EDNRB) (16). Endothelins play critical roles in regulating vascular homeostasis, such as atherosclerosis and pulmonary hypertension (17). Endothelins are also implicated vascular diseases of several organ systems, including the heart, lungs, kidneys, and brain (18). Endothelin 1 has been reported to promote SMC migration and is critical for neointima hyperplasia in giant-cell arteritis (19). Endothelin 1 also contributes to regulate vascular remodeling (20). However, the underlying mechanism of endothelin 1 in regulating pathological vascular remodeling is not well defined.

Traditional Chinese Medicine is used in the treatment of cardiovascular diseases. Numerous studies indicated that Andrographolide (21) suppresses vascular angiogenesis through p300, VEGF, and Mir-21-5p/TIMP3 signaling pathways (22–25). Andrographolide inhibits neointimal hyperplasia in arterial restenosis (26). Our previous studies demonstrated that Andrographolide is critical in gastric vascular homeostasis regulation (27). However, the role of Andrographolide in regulating pathological vascular remodeling through SMC phenotypic switching has not been reported. We found that Andrographolide promoted SMC contractile-specific marker gene expression in different culture conditions, as well as *in vivo* vascular injury studies. We observed that Andrographolide significantly promotes the expression of EDNRA, EDNRB, SRF, and Myocardin *in vivo* and *in vitro*. However, whether and how Andrographolide can regulate pathological vascular remodeling still need to be illustrated. In this study, we tried to determine whether Andrographolide suppresses pathological vascular remodeling by enhancing the interaction between EDNRA, EDNRB, and Myocardin-SRF to regulate smooth muscle cell differentiated marker gene expression.

## Materials and Methods

### Animal Ethical Approval

The use of mice in this study was approved by the Experimental Animal Ethics Committee of Chengdu University of Traditional Chinese Medicine. Ethical approval number: 2019-04.

### Mouse Common Carotid Artery Complete Ligation Model

The mouse left common carotid artery complete ligation model that induces vascular remodeling is based on the previously described method (28). Briefly, mice were pretreated with Andrographolide (10 mg/kg) for 5 consecutive days, and anesthetized with ketamine (80 mg/kg) and xylazine (5 mg/kg) by intraperitoneal injection. We exposed the left common carotid arteries and completely ligated them at the bifurcation site with 6-0 silk. The right carotid artery was exposed, but not ligated. After continuous consecutive treatment with Andrographolide for 14 or 21 days, sections (5 μm) were collected between 100 and 1,000 μM away from the ligation site. Morphological analysis based on H&E staining was conducted. The quantification of neointima areas and media layer area was completed using Image J software (29).

### Rat Aortic SMC Culture

SMC culture from the thoracic artery of Sprague-Dawley rats was separated as previously reported (30, 31). Briefly, we harvested thoracic aorta after the rats were anesthetized, removed periadventitial tissues, and denuded the endothelium under a microscope. We digested the aorta with a Blend enzyme III solution (Roche, 0.5 U/ml) for 10 min at 37°C, and removed the adventitial layer. Then, we minced the medial layer into small pieces, and following a second digestion with Blend enzyme III for 2 h at 37°C, we suspended cells in 10% FBS DMEM medium.

### CCK8 Cell Proliferation Assay

A total of 3 × 10^3^ rat SMCs (each well) were seeded in a 96-well culture plate, and treated with Andrographolide (5 μM) for 24 h. Absorbance at 450 nm was evaluated using a CCK8 kit.

### BRDU Incorporation Assay

Rat SMCs were suspended in culture media contained Andrographolide (5 μM), followed by BRDU reagent labeling for 24 h. Immunofluorescence staining was performed to determine BRDU-incorporated SMCs.

### Scratch Wound Healing Assay

The rat SMCs were seeded into a 6-well culture dish. Scratch wounds were made with a 10 μl pipette tip, and scratch gaps were monitored at different time points based on crystal violet staining.

### Boyden Chamber Migration Assay

A total of 1 × 10^6^ rat SMCs were suspended in 100 ul of FBS free culture media and seeded in a Boyden chamber (353097, FALCON). We set up the Boyden chamber with a 24-well culture plate which contained 500 μl of complete culture medium (10% FBS) and 5 μM Andrographolide. We fixed the cells after incubation for 12–24 h, and manually counted cells numbers in five random microscopic fields after crystal violet staining (32).

### Spheroid Sprouting Assay

The spheroid sprouting assay was performed as described previously (33). The methylcellulose solution was prepared by dissolving 6 g of methylcellulose (sigma) into 250 ml of prewarmed serum free medium, and 250 ml of DMEM containing 10% serum was added. Suspended cells were added to the dissolved methylcellulose solution which was prepared by 10 ml of methylcellulose solution and 40 ml of culture medium to form the spheres. We added the neutralized collagen solution to a 24-well culture plate and incubated it at 37°C until the collagen solidified. We mixed the spheres with dissolved collagen solution and transferred it to a collagen-solidified culture plate. We solidified the culture plate for 30 min at 37°C, added 200 ul of complete medium containing Andrographolide, and cultured overnight. Spheroid sprouting was visualized after calcein AM staining. Images were captured using a confocal microscope (Leica Microsystem CMS GmbH). The number of sprouts and the sprout length of each sphere were analyzed by Image J software.

### Co-immunoprecipitation Assay

Total protein from rat SMCs was extracted using RIPA buffer. We precleared the cell lysate using anti-species-specific IgG beads. We incubated the precleared cell lysate with EDNRB (abcam), EDNRA (abcam), Myocardin (Santa Cruz), and SRF (abcam) for 1 h at 4°C. Next, we incubated the lysate with pre-equilibrated protein A/G agarose beads on a rocking platform overnight at 4°C. The co-immunoprecipitated targets were evaluated by western blotting.

### SiRNA Transfection

Scrambled siRNA and siRNA targeting rat SRF and EDNRA were synthesized from GenePharma. The siRNAs were transfected into rat SMCs by using Lipofectamin 2000 reagent following the manufacturer's protocol.

### Quantitative Real Time PCR Analysis

Total RNA from rat SMCs was extracted using Trizol reagent. Quantification of RNA was monitored by a spectrophotometer (Denovix, USA). A total of 600 ng of RNA was used as the template, random hexamer primers were used for the reverse transcription reaction to obtain cDNA using an iScript cDNA synthesis kit. Real-time PCR was performed twice for each sample on the Bio-Rad real-time PCR system. The primer sequences used in this study are exhibited in Table 1. The relative gene expression level was analyzed using the 2^−ΔΔct^ method against RPLP0.

**Table 1:** List of primer sequences used for real time PCR in the study.

**Gene name**	**Species**	**Sequence**
RPLPO	Rat	F: 5′-GGACCCGAGAAGACCTCCTT-3 ′
	Rat	R: 5′-TGCTGCCGTTGTCAAACACC-3′
SRF	Rat	F: 5′-GATGGAGTTCATCGACAACAAGCTG-3′
	Rat	R: 5′-CCCTGTCAGCGTGGACAGCTCATA-3′
SM α-actin	Rat	F: 5′-ATGCTCCCAGGGCTGTTTTCCCAT-3′
	Rat	R: 5′-GTGGTGCCAGATCTTTTCCATGTCG-3′
Calponin	Rat	F: 5′-AACTGGCACCAGCTGGAGAACATAG-3′
	Rat	R: 5′-GAGTAGACTGAACTTGTGTATGATTGG-3′
SM MHC	Rat	F: 5′-CAGTTGGACACTATGTCAGGGAAA-3′
	Rat	R: 5′-ATGGAGACAAATGCTAATCAGCC-3′
Myocardin	Rat	F: 5′-GTTCAGCTACCCTGGGATGCACCAA-3′
	Rat	R: 5′-GGCCTGGTTTGAGAGAAGAAACACC-3′
KLF4	Rat	F: 5′-CGGGAAGGGAGAAGACACTGC-3′
	Rat	R: 5′-GCTAGCTGGGGAAGACGAGGA-3′
SM22α	Rat	F: 5′-TGACATGTTCCAGACTGTTGACCTCT-3′
	Rat	R: 5′-CTTCATAAACCAGTTGGGATCTCCAC-3′
MRTFA	Rat	F: 5′-CAGAGAGATCAGAGCTGGTCAG−3′
	Rat	R: 5′-CATCGCTGCTGTCCTCGTCAAA-3′
FGF9	Rat	F: 5′-GACTTGCCGATTTGCTCTGCACTT-3′
	Rat	R: 5′-AGCCTCTCTCCCTGCTTTCACAAT-3′
SIRT1	Rat	F: 5′-TAGCCTTGTCAGATAAGGAAGGA-3′
	Rat	R: 5′-ACAGCTTCACAGTCAACTTTGT-3′
Gata6	Rat	F: 5′-GCCCCTCATCAAGCCACA−3′
	Rat	R: 5′-CATAGCAAGTGGTCGAGGCA−3′
TBX2	Rat	F: 5′-CATCCTGAACTCCATGCACAAG−3′
	Rat	R: 5′-ACAGTGCTCCTCATACAAACGG−3′
TBX3	Rat	F: 5′-TTATAGCTGCTGATGACTGTCG−3′
	Rat	R: 5′-GCTGGTACTTGTGCATGGAGTT−3′
TBX18	Rat	F: 5′-CGAGTGCACATCATCCGTAAAG−3′
	Rat	R: 5′-GCATATGACTCCACCAGAGCTT−3′
EDN1	Rat	F: 5′-GACCAGCGTCCTTGTTCCAA-3′
	Rat	R: 5′-TTGCTACCAGCGGATGCAA-3′
EDN2	Rat	F: 5′-GGCTTGACAAGGAATGTGTGTACT-3′
	Rat	R: 5′-CACGTCTTGCTAGTCTCTAACACA-3′
EDN3	Rat	F: 5′-CGTGCTTCACTTATAAGGACAAGG-3′
	Rat	R: 5′-CAACGTAAGCGTGTCTGTGGAGAA-3′
EDNRA	Rat	F: 5′-CTCAACGCCACGACCAAGTT-3′
	Rat	R: 5′-GCAAGCTCCCATTCCTTCTG-3′
EDNRB	Rat	F: 5′-TGGCCATTTGGAGCTGAGAT-3′
	Rat	R: 5′-TCCAAGAAGCAACAGCTCGAT-3′
CCND1	Rat	F: 5′-AATGGAACTGCTTCTGGTGAACA-3′
	Rat	R: 5′-CGGATGATCTGCTTGTTCTCATC-3′
c-Myc	Rat	F: 5′-CCCCTCAGTGGTCTTCCCCTAC-3′
	Rat	R: 5′-TGTTCTCGCCGTTTCCTCAGTA-3′
ADK	Rat	F: 5′-TGGCTTCTTTCTCAGCGTCT-3′
	Rat	R: 5′-ACTCCACAGCCTGAGTTGCT-3′
CDKN1A	Rat	F: 5′-ATGACTGAGTATAAACTTGTGG-3′
	Rat	R: 5′-TCAVATGACTATACACCTTGTC-3′
CDKN1B	Rat	F: 5′-GTCTCAGGCAAACTCTGAG-3′
	Rat	R: 5′-GTTTACGTCTGGCGTCGAAG-3′
p53	Rat	F: 5′-GACTTCTTGTAGATGGCCATGG-3′
	Rat	R: 5′-ATGGAGGATTCACAGTCGGATA-3′
GADD45	Rat	F: 5′-ATGACTTTGGAGGAATTCTCGG-3′
	Rat	R: 5′-TCACCGTTCGGGGAGATTAATC-3′
PTEN	Rat	F: 5′-GCACAAGAGGCCCTGGATT-3′
	Rat	R: 5′-TGAAACAACAGTGCCACTGG-3′
c-Fos	Rat	F: 5′-GGGACAGCCTTTCCTACTACC-3′
	Rat	R: 5′-AGATCTGCGCAAAAGTCCTG-3′
IL-15	Rat	F: 5′-ACTACCTGTGTTTCCTTCTCAAC-3′
	Rat	R: 5′-TTGGCCTCTGTTTTAGGG-3′
IL-18	Rat	F: 5′-TCCTTTGAGGAAATGAATCC-3′
	Rat	R: 5′-GCTAGAAAGTGTCCTTCATAC-3′
PDCD4	Rat	F: 5′-AACTATGATGATGACCAGGAGAAC-3′
	Rat	R: 5′-GCTAAGGACACTGCCAACAC-3′
CCN3	Rat	F: 5′-GGAAGTGCATTCGTTGAGGC-3′
	Rat	R: 5′-AAGCAAGTCACCCCTAAGCC-3′
MyD88	Rat	F: 5′-GATCCCACTCGCAGTTTGTT-3′
	Rat	R: 5′-GATGCGGTCCTTCAGTTCAT-3′
Thbs-1	Rat	F: 5′-CGGTTTGATCAGAGTGGTGA-3′
	Rat	R: 5′-CGGCACTCGTATTTCATGTC-3′
Cdh13	Rat	F: 5′-AACCCACAGACCAACGAG-3′
	Rat	R: 5′-TGATCAGCAGGGTGTGAA-3′
Hif1α	Rat	F: 5′-ACAGGATTCCAGCAGACCC-3′
	Rat	R: 5′-GCTGATGCCTTAGCAGTGGTC−3′
IGFBP5	Rat	F: 5′-ATGAAGCTGCCGGGC-3′
	Rat	R: 5′-TCAACGTTACTGCTGTCGAAG-3′
IGF1	Rat	F: 5′-GGCACTCTGCTTGCTCACCTTT-3′
	Rat	R: 5′-CACGAATTGAAGAGCGTCCACC-3′
GSK3β	Rat	F: 5′-GGGCACCAGAGCTGATCTTT-3′
	Rat	R: 5′-GCCGAAAGACCTTCGTCCA-3′
Cav1	Rat	F: 5′-GACGAGGTGAATGAGAAGCAAG-3′
	Rat	R: 5′-GAGAGGATGGCAAAGTAGATGC-3′
Ddx39b	Rat	F: 5′-CAACTATGACATGCCAGAGGAC-3′
	Rat	R: 5′-GATTCCTCTACCGTGTCTGTTC-3′

### Protein Extraction and Western Blotting

Protein from rat SMCs was extracted using RIPA lysis buffer. Protein concentration was determined by a BCA kit (Biosharp). The protein was denatured at 98°C, separated by sodium dodecyl sulfate-polyacrylamide gel electrophoresis (SDS-PAGE), and transferred onto polyvinylidene fluoride (PVDF) membranes. Next, we blocked the protein with 5% fat free milk, and incubated it with specific antibodies at 4°C overnight. Images were captured using an ImageQuant LAS 4000 Imager Station and we quantified densities of protein bands using ImageQuant TL software.

### Hematoxylin and Eosin Staining, Immunohistochemistry, and Immunofluorescence Staining (IF)

We harvested carotid arteries and fixed them with 4% paraformaldehyde overnight at 4°C. Slides of 5 μm thickness were collected after being paraffin-embedded. H&E staining was performed as per our previous study (34). For IHC staining, the slides were deparaffinized, antigen retrieval was performed by citric acid treatment at 98°C for 5 to 10 min. After antigenic unmasking, the slides were incubated with EDNRB (abcam), EDNRA (abcam), Myocardin (Santa Cruz), and SRF (abcam) overnight at 4°C, followed by incubation with biotinylated secondary antibody at room temperature for 1 h (Vector Laboratories), and then incubated with ABC solution (Vector Laboratories) for 30 min at room temperature. The targets were visualized after the DAB solution was added. For IF staining, the deparaffinized slides were permeabilized with PBS contained 0.25% Triton-X-100, blocked with 10 % goat serum, incubated with primary antibodies overnight at 4°C, washed with PBST, and incubated with Alexa 594-conjugated or Alexa 488-conjugated secondary antibody at room temperature for 1 h. Nuclei were visualized with 4′, 6′-diamidino-2-phenylindole (DAPI) staining. For BRDU staining, DNA was denaturized using 2N HCl, followed by antibodies incubation. Images were captured using confocal microscopy (LS510, Zeiss).

### Statistics

Quantitative data are presented as mean±SEM. The statistical analysis was performed by GraphPad prism software. Normal distribution was determined by the Kolmogorov-Smirnov test. Statistical comparisons between two groups were analyzed using two-tailed unpaired Student's *t* test or one- or two-way analysis of variance (ANOVA) followed by Bonferroni's *post hoc* tests when appropriate. Two-sided *P* values were quantified. ^*^ *P* < 0.05 was considered statistically significant.

## Results

### Andrographolide Attenuates Neointima Hyperplasia Induced by Vascular Ligation Injury

**Figure 1:**
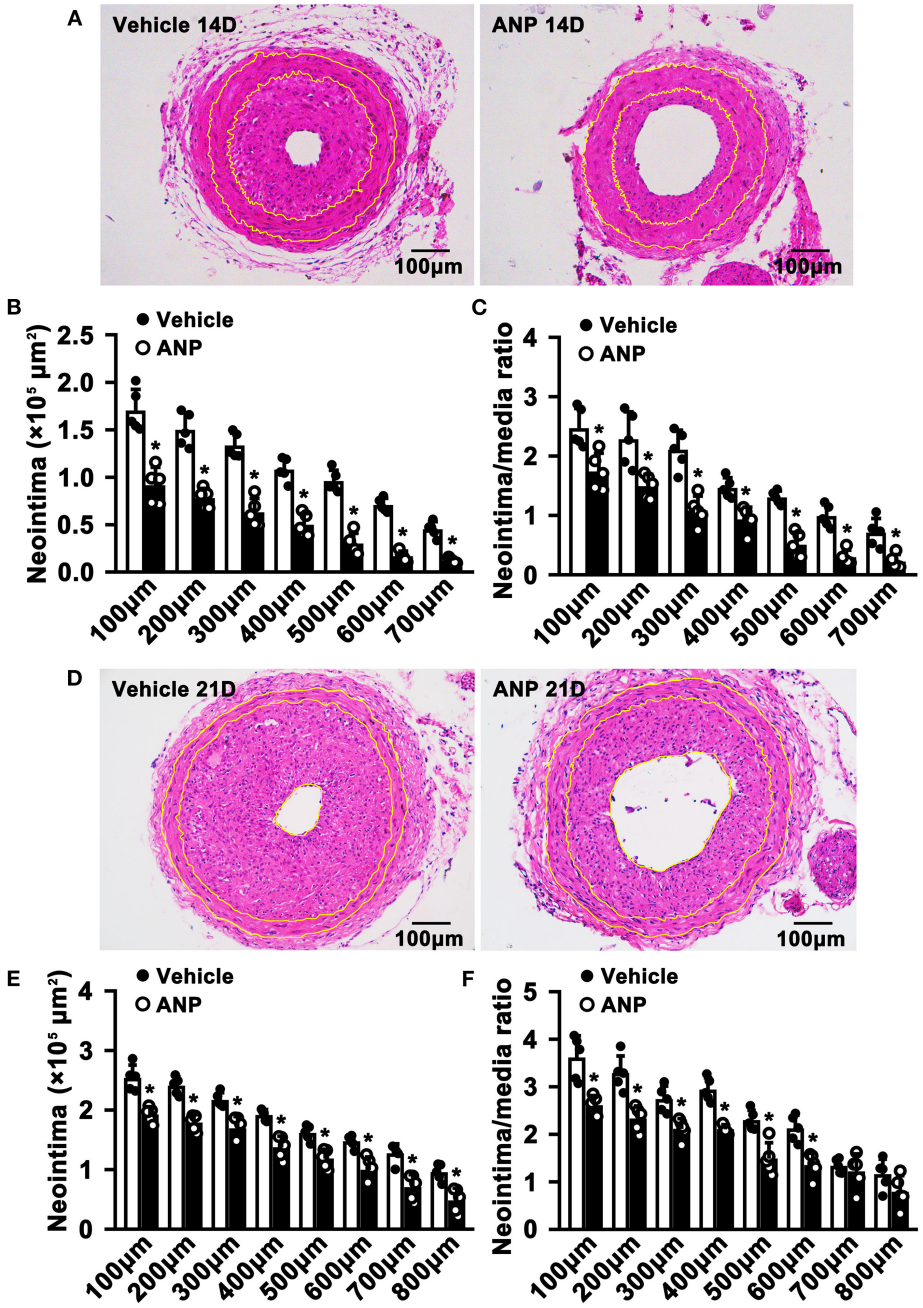
Andrographolide attenuates neointima hyperplasia induced by vascular ligation injury. **(A)** Complete ligation of the left common carotid artery in C57BL/6 mice, and following Andrographolide treatment (10 mg/kg) by intraperitoneal injection for 14 consecutive days. The arteries were harvested and paraffin-embedded. Slides of 5 μm thickness at different locations from the ligation site were collected. H&E staining was performed to visualize vascular morphological changes. **(B,C)** Analysis areas of the neointimal hyperplasia and ratio of the neointimal areas to the medium layer area (*n* = 5). **(D)** Andrographolide (10 mg/kg) was administered by intraperitoneal injection for 21 consecutive days, the representative image of H&E staining. **(E,F)** Analysis areas of the neointimal hyperplasia and ratio of the neointimal areas to the medium layer area (*n* = 5). Data are expressed as mean ± SEM. *P< *0.05*.

To evaluate whether Andrographolide had an inhibitory effect on neointimal hyperplasia, we created a vascular injury model using C57BL/6 mice and complete ligation of the left common carotid artery, followed by consecutive Andrographolide treatment (10 mg/kg) (21, 27). The arteries were harvested and paraffin-embedded. Slides of 5 μm thickness at different locations from the ligation site were collected. We performed H&E staining to visualize vascular morphological changes. After 14 consecutive days of treatment, Andrographolide significantly attenuated neointimal hyperplasia (Figure 1A). We analyzed the neointima areas at different locations from the ligation site using Image J software. Our data showed that the neointimal areas from 100 to 700 μm distance significantly decreased after Andrographolide treatment (Figure 1B). We compared the ratio of neointima area to media smooth muscle layer area, and found the ratio significantly deceased after Andrographolide treatment (Figure 1C). The areas of the media smooth muscle cells layer were determined, however, no significance was found (Supplementary Figure 1A). We also observed the vascular morphological changes after 21 consecutive days of treatment with Andrographolide, our data demonstrated that Andrographolide treatment significantly inhibited neointimal hyperplasia (Figure 1D), decreased both neointima areas and the ratio of neointima area to media smooth muscle layer area (Figures 1E,F), whereas no statistical changes were observed on the media smooth muscle cell layer (Supplementary Figure 1B). The data indicated that Andrographolide significantly attenuated the formation of vascular neointimal hyperplasia.

### Andrographolide Is Critical in Regulating Smooth Muscle Cell Phenotypic Switching

**Figure 2:**
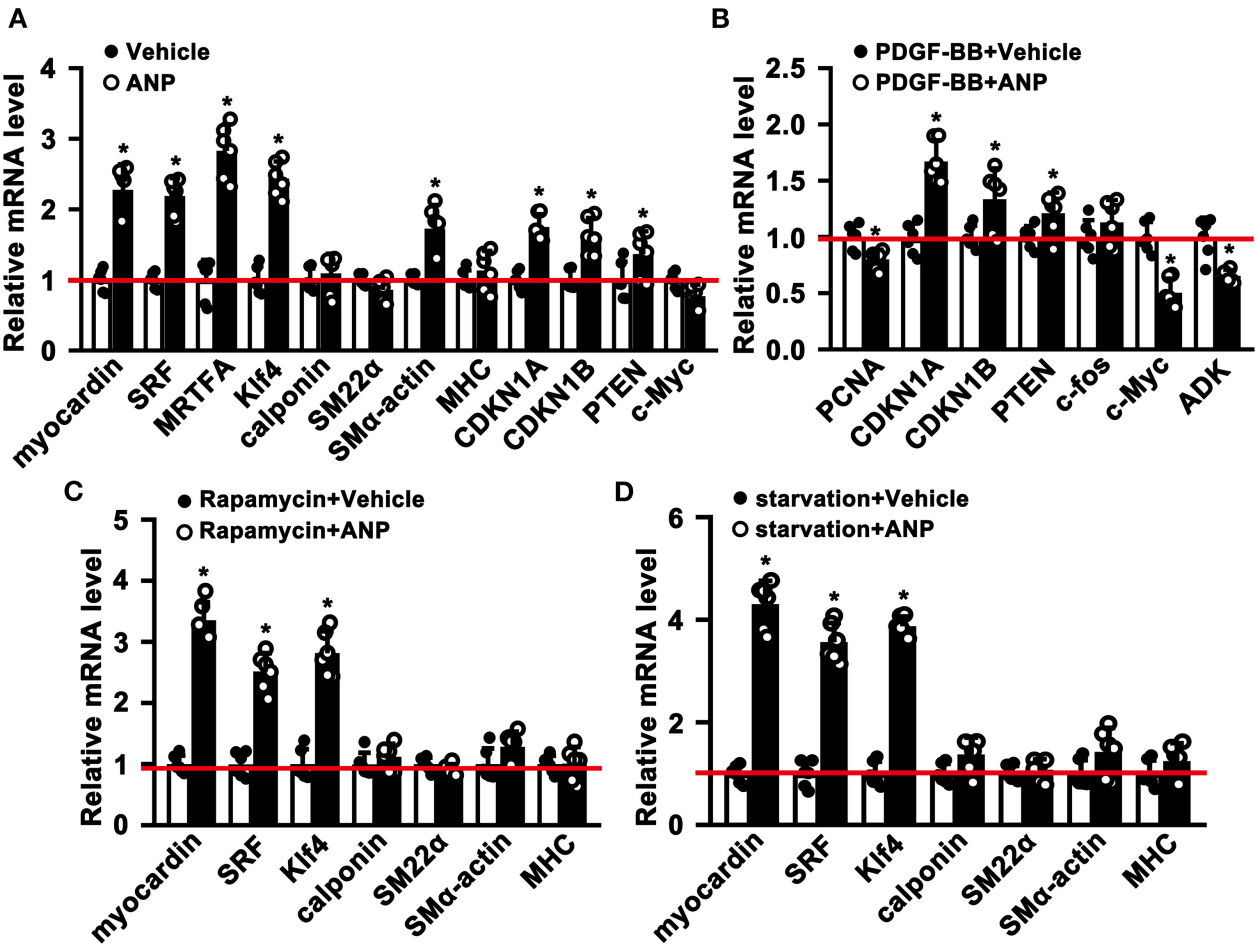
Andrographolide is critical in regulating smooth muscle cells phenotypic switching. **(A)** Rat SMCs were treated with Andrographolide (5 μM) for 30 h, the mRNA levels of SMC-specific marker genes, including myocardin, SRF, klf4, calponin, SM22α, SM α-actin, MHC, MRTFA, and proliferation-related genes including CDKN1A, CDKN1B, PTEN, and c-Myc were detected by real-time PCR (n = 6). **(B)** Proliferation of rat SMCs induced by PDGF-BB (25 ng/ml) incubation, following Andrographolide (5 μM) treatment. Proliferation-related genes were evaluated by real-time PCR (*n* = 6). **(C)** Differentiation of rat SMCs was induced by rapamycin (100 nM/L), following Andrographolide (5 μM) treatment. Real-time PCR was performed to determine SMC-specific marker gene expression (*n* = 6). **(D)** Differentiation of rat SMCs was mimicked by starvation (0.2% FBS), following Andrographolide (5 μM) treatment. Real-time PCR was performed to determine SMC-specific marker gene expression (*n* = 6). Data are expressed as mean ± SEM. *P < 0.05.

The phenotypic switching of SMCs plays a critical role during the process of pathological vascular remodeling. However, whether Andrographolide is critical in regulating SMC phenotypic switching is not well defined. We cultured primary rat aortic SMCs and treated them with Andrographolide (5 μM). After treatment for 30 h, quantitative real-time PCR was performed to determine the transcription level of SMC differentiated genes and cell growth positive regulated genes. The data indicated that SMC differentiated genes, including Myocardin, SRF, MRTFA, klf4, and smooth muscle α-actin were dramatically increased, and genes inhibiting cell proliferation, such as CDKN1A and CDKN1B, were upregulated (Figure 2A). Some other signaling pathways involved in SMC differentiation regulation were also enhanced (Supplementary Figure 2A). The characteristics of phenotypic switching for matured SMCs exhibited enhanced proliferation, whereas differentiation was decreased. We sought to determine whether Andrographolide is critical in regulating SMC phenotypic switching. First, we mimicked a condition that promotes cell growth by PDGF-BB treatment (Supplementary Figure 2B). With presence of PDGF-BB, Andrographolide treatment could inhibit the expression of PCNA, c-Myc, and ADK, whereas it enhanced CDKN1A, CDKN1B, and PTEN expression (Figure 2B). We next induced SMC differentiation by Rapamycin treatment (Supplementary Figure 2C). After Rapamycin incubation, following Andrographolide treatment, SMC differentiated specific marker genes, including Myocardin, SRF, KLF4, Calponin, SM22a, and MHC markedly induced differentiation (Figure 2C). We further induced SMC differentiation by starvation treatment (Supplementary Figure 2D). Andrographolide treatment also promoted the expression of Myocardin, SRF, KLF4, Calponin, and smooth muscle a-actin (Figure 2D). Our data demonstrated that Andrographolide is critical in regulating SMC phenotypic switching.

### Andrographolide Inhibits Proliferation of Vascular Smooth Muscle Cells

**Figure 3:**
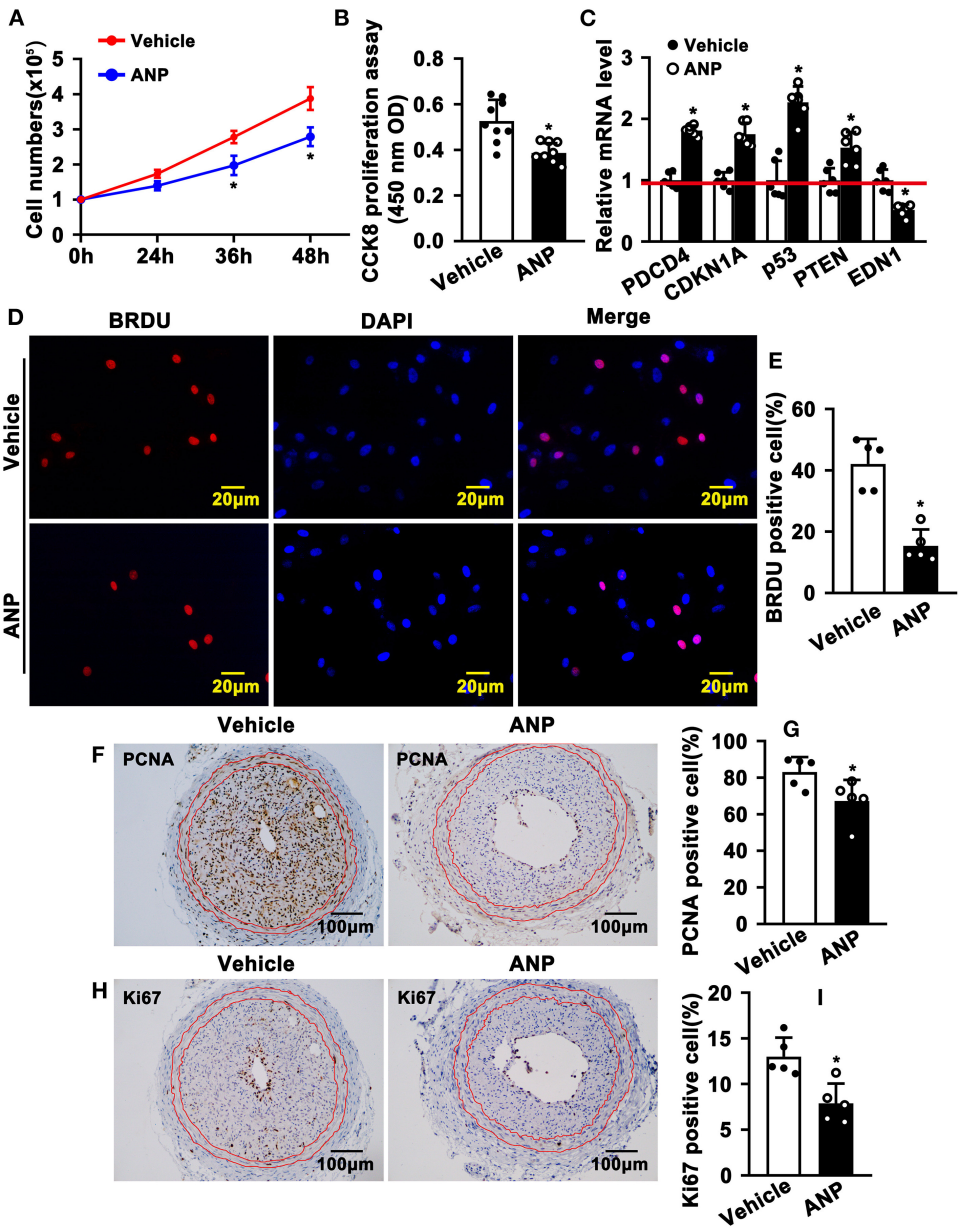
Andrographolide inhibits proliferation of vascular smooth muscle cells. **(A)** Rat SMCs were treated with Andrographolide (5 μM), and the cell numbers were counted at different time points (*n* = 6). **(B)** Cell viability was detected by a CCK8 cell proliferation assay (*n* = 8). **(C)** The mRNA levels of proliferation-related genes were detected by real-time PCR (*n* = 6). **(D)** Rat SMCs were incubated with BRDU labeling buffer for 20 h, following Andrographolide treatment overnight. Immunofluorescence staining was performed to evaluate BRDU incorporation and BRDU-positive cells shown in **(E)**. **(F,H)** Immunohistochemical staining was performed against proliferation marker genes PCNA and Ki67 on the left common carotid artery complete ligation model. PCNA and Ki67-positive SMCs in neointimal areas are shown in **(G,I)** (*n* = 5). The analysis data are expressed as means ± SEM. *P < 0.05.

The hallmark of SMC phenotypic switching is characterized by enhanced proliferation. We sought to determine whether Andrographolide can inhibit SMC proliferation. We treated rat aortic SMCs with Andrographolide (5 μM) and the cell numbers were counted. Andrographolide treatment significantly decreased cell numbers at 36 h and 48 h following treatment (Figure 3A). Andrographolide treatment decreased SMCs viability which was measured by CCK8 (Figure 3B). Cell growth-related genes were determined by real-time PCR. Andrographolide treatment promoted expression of cell cycle negative-related genes, including PDCD4, CDKN1A, P53, and PTEN (Figure 3C). The BRDU incorporation assay was also performed to evaluate whether Andrographolide could suppress SMC proliferation. With the addition of Andrographolide, the BRDU-positive cells—which were detected by immunofluorescence staining—were dramatically decreased (Figures 3D,E). That Andrographolide suppressed SMC proliferation was validated *in vivo*. We performed immunohistochemistry staining on slides from injured animals. Andrographolide treatment dramatically decreased the number of both PCNA and Ki67 positive numbers within the neointimal area (Figures 3F–I). However, no statistical difference of PCNA or Ki67 positive numbers within the media smooth muscle layer between the Andrographolide treatment group and vehicle treatment group was exhibited (Supplementary Figures 3A,B). The data demonstrated that Andrographolide suppressed SMC proliferation.

### Andrographolide Inhibits Migration of Rat Smooth Muscle Cells

**Figure 4:**
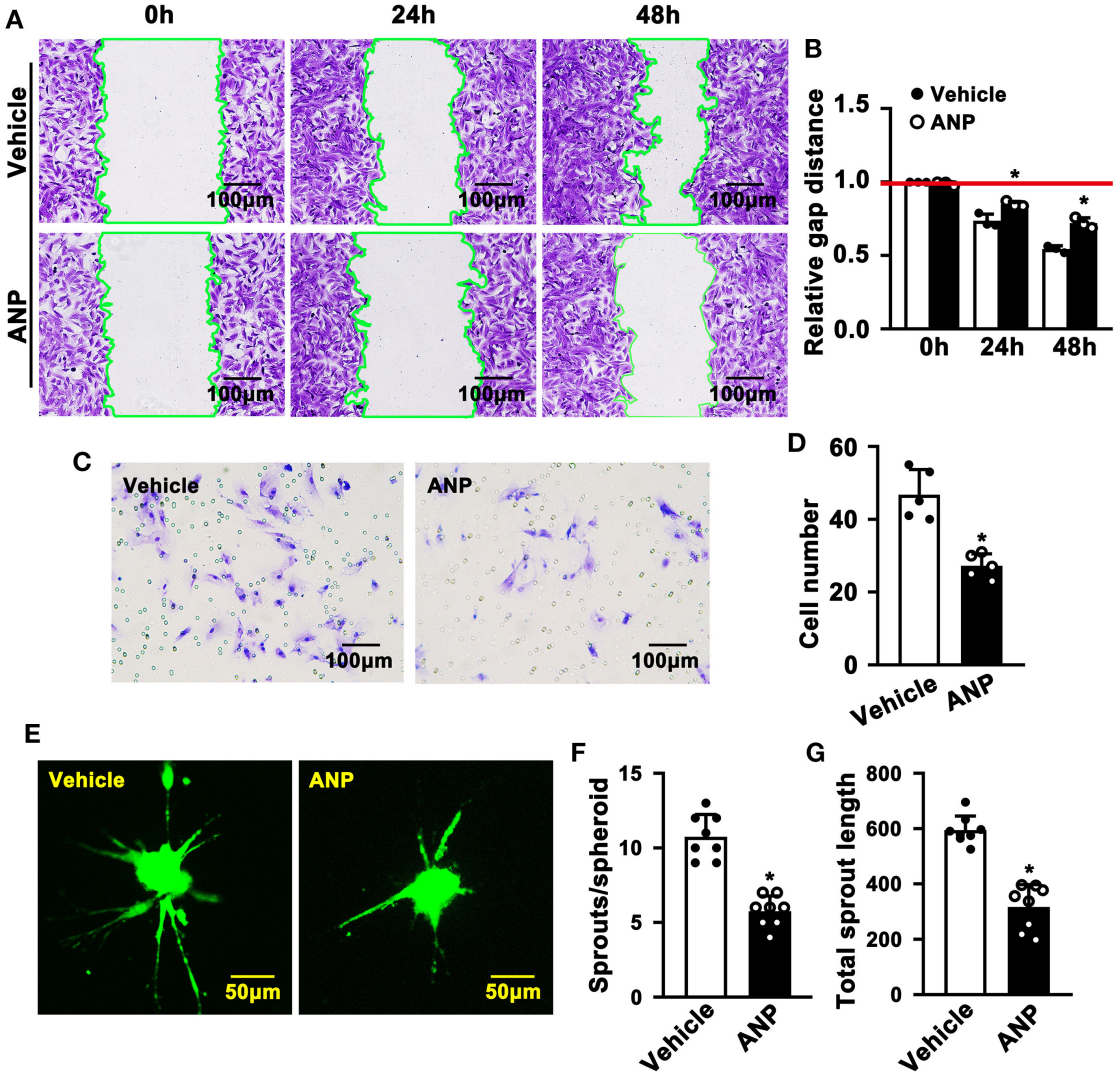
Andrographolide inhibits migration of rat smooth muscle cells. **(A)** A wound scratching assay was performed to determine whether Andrographolide treatment (5 μM) suppressed SMC migration. The number of scratch gaps at 24 h and 48 h is exhibited in **(B)** (*n* = 5). **(C)** A Boyden chamber cell migration assay was performed in the presence of Andrographolide (5 μM), the migrated cells were visualized by crystal violet staining, and cell numbers are exhibited in **(D)** (*n* = 5). **(E)** A spheroid sprouting assay was performed in the presence of Andrographolide (5 μM), the sprouting of SMCs was visualized by calcein AM staining. Quantification of sprouts and sprout length is exhibited in **(F,G)** (*n* = 8). The analysis data are expressed as means ± SEM. *P < 0.05.

Enhanced migration of smooth muscle cells is evident during SMC phenotypic switching. To explore whether Andrographolide plays a critical role in regulating the migration of SMC, we performed a scratch wound healing assay following crystal violet staining to visualize the scratch gap at different time points. Much bigger scratch gaps were seen after Andrographolide treatment (Figures 4A,B). We next performed a Boyden chamber migration assay to evaluate the effect of Andrographolide on regulating the migration of SMCs. The data indicated that Andrographolide treatment observably reduced rat SMCs passing through the Boyden chamber (Figures 4C,D). Furthermore, we performed a spheroid sprouting assay, and found that Andrographolide remarkably suppressed both sprouts and sprout length (Figures 4E–G). The data indicated that Andrographolide could significantly inhibit the migration of rat SMCs.

### Andrographolide Activates Endothelin Family Response to Vascular Injury Stress

**Figure 5:**
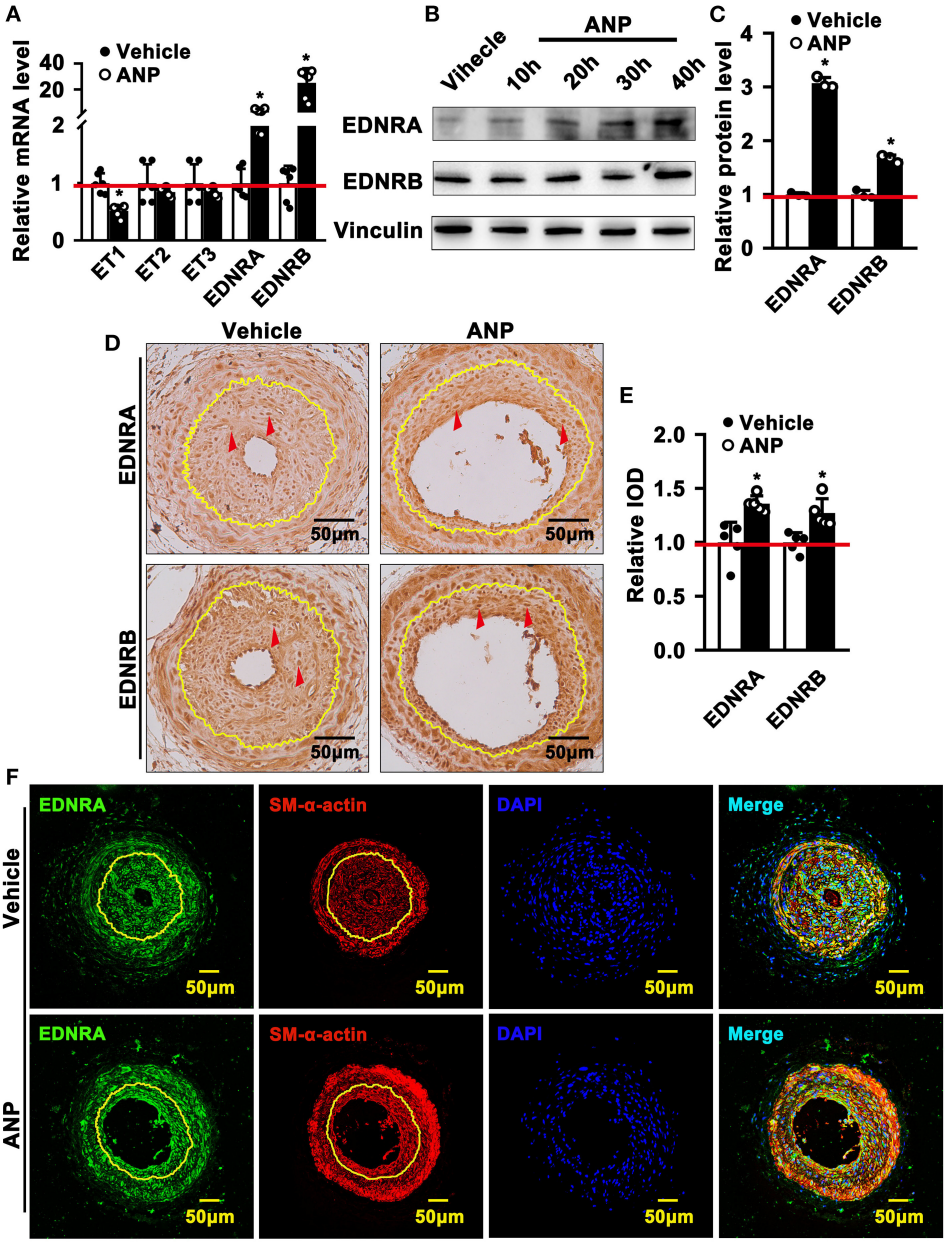
Andrographolide activates endothelin family response to vascular injury stress. **(A)** Rat SMCs were treated with Andrographolide (5 μM) for 30 h. Real-time PCR was performed to detect the mRNA level of the endothelin family (*n* = 6). **(B)** Western blot was performed to determine the expression of EDNRA and EDNRB in SMCs after Andrographolide (5 μM) treatment for different time points, and the quantification data are shown **(C)** (*n* = 3). **(D)** Immunohistochemistry staining was performed to detect the expression of EDNRA and EDNRB in the completely ligated carotid arteries. **(E)** The relative protein levels were quantified by Average Optical Density (Integrated option density/Area) using Image J software (*n* = 5). **(F)** Immunofluorescence staining was used to evaluate the expression of EDNRA ligated left carotid arteries. The analysis data are expressed as means ± SEM. *P < 0.05.

Andrographolide plays a critical role in maintaining the differentiated stage of SMCs which is characterized by inhibiting proliferation and migration of vascular SMCs. However, the underlying mechanism is not well defined. We performed real-time PCR to evaluate different signaling pathways that are associated with SMC phenotypic switching regulation. The data indicated that expression of Myd88, P53, IGFBP5, GS3Kβ, EDNRA, and EDNRB was dramatically enhanced after Andrographolide treatment. The most obvious change in expression level occurred in EDNRA and EDNRB (Supplementary Figure 4). Both EDNRA and EDNRB are receptors for the endothelin/sarafotoxin family which is critical in regulating vasoconstriction. We first evaluated the expression of ET1, ET2, and ET3 in SMCs. We observed the highest expression level in ET1 under normal culture conditions (Supplementary Figure 5A). The expression of EDNA was much higher than that of EDNB (Supplementary Figure 5B). In order to confirm the potential targets for Andrographolide, we further treated rat SMCs with different doses of Andrographolide, and performed real-time PCR to determine the expression of the endothelin family and its receptors. Treatment with 1 μM Andrographolide did not change the expression of ET1, ET2, and ET3 in rat SMCs, whereas it significantly induced EDNRA and EDNRB expression (Supplementary Figures 6A,B). The treatment with 5 μM Andrographolide dramatically suppressed the transcription levels of ET1, ET2, and ET3 in rat SMC, whereas it observably induced EDNRA and EDNRB transcription levels (Figure 5A). The protein levels of EDNRA and EDNRB were also enhanced after 5 μM Andrographolide treatment in SMCs (Figures 5B,C). We induced proliferation of rat SMCs by PDGF-BB treatment, and similar results were exhibited (Supplementary Figures 6C,D). To validate whether Andrographolide could regulate the endothelin/sarafotoxin family *in vivo*, we performed immunohistochemistry against EDNRA and EDRNB antibodies on pathological sections of complete ligation of the left common carotid artery. The results showed that the expressions of EDNRA and EDRNB were remarkably increased after Andrographolide treatment (Figures 5D,E). That Andrographolide treatment enhanced the expression of EDNRA was also validated by immunofluorescence staining (Figure 5F). The data demonstrated that Andrographolide activates the endothelin/sarafotoxin family by increasing both EDNRA and EDRNB in vascular SMCs.

### Andrographolide Promotes the Interaction of EDNRA and EDNRB and the Myocardin-SRF Complex Resulting in Inducing the Expression of SMC Differentiated-Specific Genes

**Figure 6:**
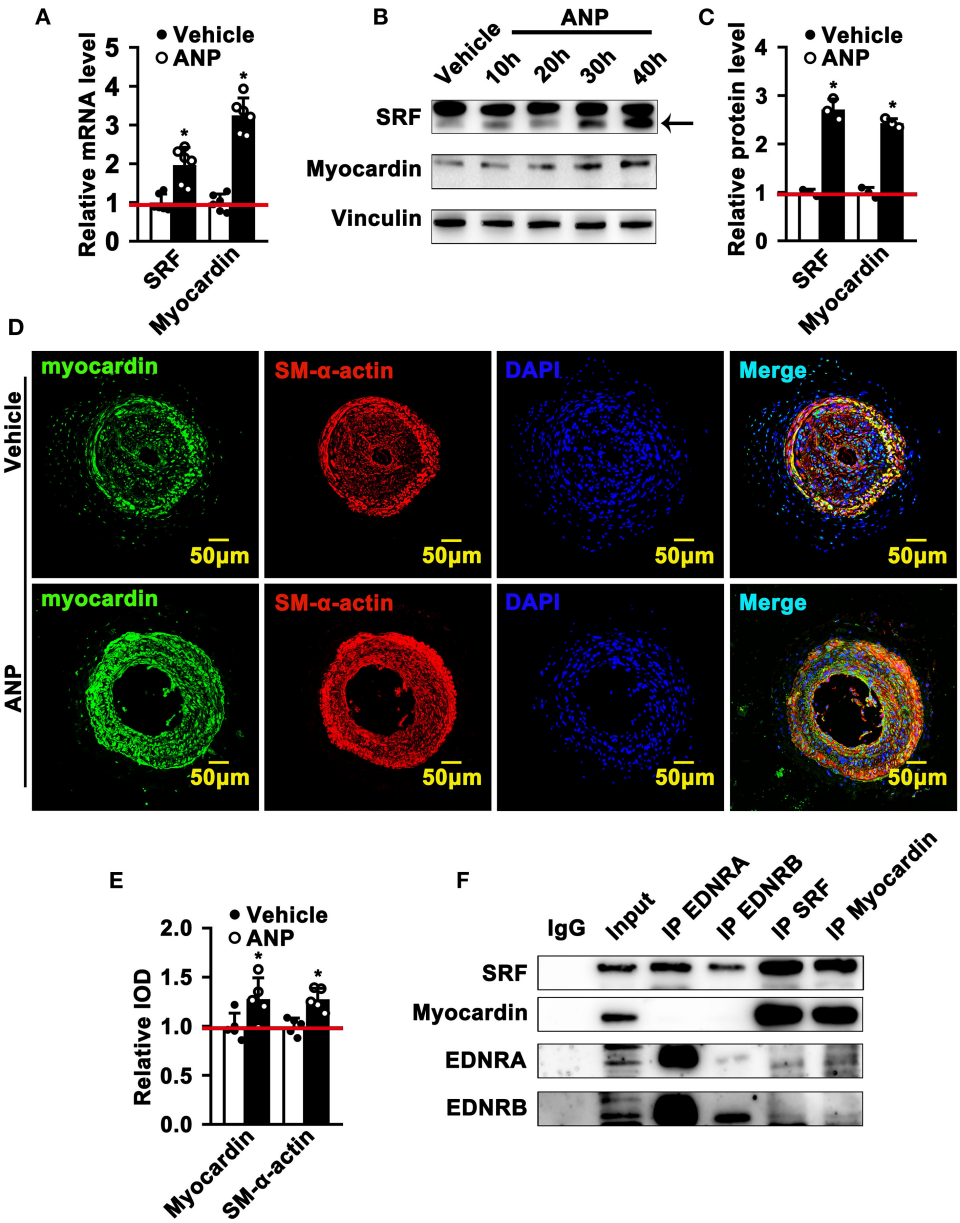
Andrographolide promotes the interaction of EDNRA and EDNRB with the Myocardin-SRF complex. **(A)** Real-time PCR was performed to determine SRF and Myocardin mRNA levels in SMCs after 5 μM Andrographolide treatment (*n* = 6). **(B)** Rat SMCs were treated with Andrographolide (5 μM) over different time points. A western blot assay was performed to evaluate the protein level of SRF and Myocardin, quantified densities of protein were bound by Integral Optical Density (IOD) using ImageQuant TL software **(C)** (*n* = 3). **(D,E)** Immunofluorescence staining was performed to evaluate the expression of Myocardin in ligated left carotid arteries. **(F)** Co-immunoprecipitation was performed to determine the interaction of EDNRA/EDNRB and Myocardin-SRF. Total protein from rat SMCs was extracted using RIPA buffer. The cell lysate was precleared using anti-species-specific IgG beads. The precleared cell lysate was incubated with EDNRB (abcam), EDNRA (abcam), Myocardin (Santa Cruz), and SRF (abcam) for 1 h at 4°C. Following incubation with pre-equilibrated protein A/G agarose beads on a rocking platform overnight at 4°C, the co-immunoprecipitated targets were evaluated by western blotting. *P < 0.05.

The most exciting and significant advance in SMC phenotypic switching is the discovery of SRF and Myocardin, which can bind to the CArGA box to regulate the expression of SMC differentiated genes (1). Andrographolide promotes the expression of both EDNRA and EDNRB in SMCs. However, whether enhanced EDNRA and EDNRB expression can regulate the expression of SRF and Myocardin is not fully defined. We first determined whether Andrographolide treatment could regulate the expression of SRF and Myocardin. We found that different doses (1 μM and 5 μM) of Andrographolide treatment dramatically enhanced SRF and Myocardin transcription levels (Supplementary Figure 7A; Figure 6A). Similar results were found with PDGF-BB (Supplementary Figure 7B). Different time points of Andrographolide (5 μM) treatment significantly induced the protein levels of SRF and Myocardin (Figures 6B,C). We next evaluated the expression of Myocardin *in vivo*; immunofluorescence staining showed that Andrographolide treatment obviously increased the expression of Myocardin in the completely ligated left common carotid artery (Figures 6D,E). To further explore whether EDNRA and EDNRB could interact with SRF and Myocardin, a co-immunoprecipitation experiment was performed. Pooled protein from rat SMCs was used. After being precleared using anti-species-specific IgG beads, incubated with EDNRB (abcam), EDNRA (abcam), Myocardin (Santa Cruz), and SRF (abcam) antibodies, and following incubation with pre-equilibrated protein A/G agarose beads, the co-immunoprecipitated proteins were evaluated by western blotting. We observed that EDNRA can bind to EDNRB to form a complex; SRF and Myocardin can also interact with each other (Figure 6F). The data indicated that EDNRA binds to EDNRB to form a complex which interacts with Myocardin-SRF complexes.

### Inhibition of Endothelin Receptors and SRF Attenuates Andrographolide-Promoted SMC Dedifferentiation

**Figure 7:**
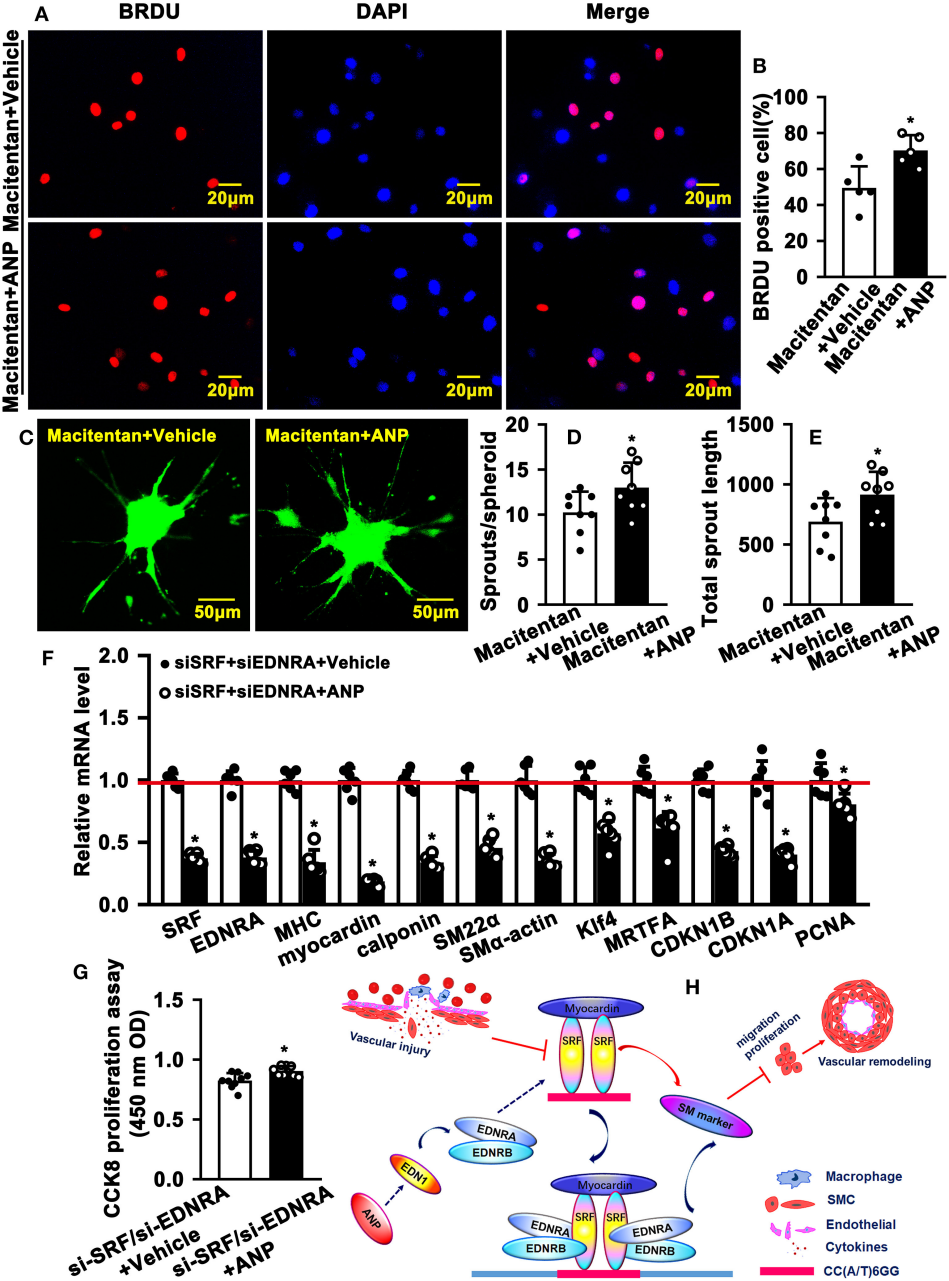
Inhibition of endothelin receptors and SRF attenuates Andrographolide-promoted SMC dedifferentiation. **(A)** Rat SMCs were treated with Macitentan (1 μM) and Andrographolide (5 μM), followed by incubation with BRDU labeling buffer for 20 h. Immunofluorescence staining was performed to observe BRDU incorporation, and BRDU-positive cells were quantified, as shown in **(B)** (*n* = 5). **(C)** A spheroid sprouting assay was performed in the presence of Macitentan (1 μM) and Andrographolide (5 μM). The sprouting was visualized by calcein AM staining, and sprouts and sprouting length are quantified in **(D,E)** (*n* = 8). **(F)** Rat SMCs were transfected with small interfering RNA si-SRF and si-EDNRA for 4-6 h, and then treated with Andrographolide (5 μM) for 30 h, the mRNA levels of SMC-specific marker genes, including SRF, Myocardin, MHC, calponin, SM22α, SMα-actin, KLF4, and MRTFA, and proliferation-related genes including CDKN1A, CDKN1B, and PCNA were detected by real-time PCR (*n* = 6). **(G)** Rat SMCs were transfected with small interfering RNA si-SRF and si-EDNRA for 4–6 h, and then treated with Andrographolide (5 μM) for 24 h, the cell viability was detected by CCK8 (*n* = 9). Data are presented as mean ± SEM. *P < 0.05. **(H)** The schematic diagram indicates that vascular injury suppresses the expression of SRF and Myocardin, resulting in decreased expression of SMC-specific marker genes, which is characterized by enhanced proliferation and migration, eventually leading to vascular hyperplasia. Treatment of SMCs with Andrographolide activates the endothelin signaling pathway and promotes the interaction of EDNA and EDNB with the Myocardin-SRF complex to induce SMC-specific marker gene expression.

EDNRA and EDNRB can bind to SRF. However, whether this binding is critical in regulating SMC phenotypic switching is not well defined. We treated SMC with Macitentan, a non-specific inhibitor for EDNRA and EDNRB, and detected whether Macitentan regulated SMC proliferation and migration. The BRDU incorporation assay and CCK8 cell proliferation assay indicated that Macitentan alone can promote SMC proliferation (Supplementary Figures 8A–C). Macitentan treatment also promoted SMC migration, which was evident in our spheroid cell migration assay (Supplementary Figures 9A–C). Following Andrographolide treatment, the BRDU incorporation assay indicated that Andrographolide suppressing SMC proliferation was obviously attenuated in the presence of Macitentan (Figures 7A,B). Similar results were exhibited in the CCK8 cell proliferation assay (Supplementary Figure 10). Macitentan dramatically attenuated the suppressed migration of SMCs induced by Andrographolide treatment (Figures 7C–E). We further used siRNA to delete EDNRA and SRF in rat SMCs. After transfection, real-time PCR was performed to evaluate deletion efficiency (Supplementary Figures 11A,B, 12A,B). The siRNA targeted deletion of EDNRA and SRF was chosen from three difference sequences, and the sequences are exhibited in Table 2. After transfection of si-EDNRA, Andrographolide treatment significantly suppressed the expression of MHC, calponin, SM22α, and smooth muscle α-actin (Supplementary Figure 13). After transfection of si-SRF, no difference in the expression of smooth muscle marker genes was found between si-SRF and Andrographolide treatment alone (Supplementary Figure 14). However, after transfection with both si-EDNRA and si-SRF, following Andrographolide treatment, the smooth muscle marker genes were dramatically inhibited, including MHC, calponin, SM22α, smooth muscle α-actin, and klf4 (Figure 7F) and which resulting the increase of cell viability (Figure 7G). The data demonstrated that Andrographolide promotes the interaction between endothelin-dependent EDNRA/EDNRB and Myocardin-SRF to regulate pathological vascular remodeling.

**Table 2:** The sequences of siRNA.

**Gene name**	**Species**	**Sequence**
Si-control	Rat	S: 5′-UUCUCCGAACGUGUCACGUTT-3′
	Rat	AS: 5′-ACGUGACACGUUCGGAGAATT-3′
Si-SRF	Rat	S: 5′-CACCUCCACAAUCCAAACATT-3′
	Rat	AS: 5′-UGUUUGGAUUGUGGAGGUGTT-3′
Si-EDNRA	Rat	S: 5′-CACGACGGCUUUCAAAUAUTT-3′
	Rat	AS: 5′-AUAUUUGAAAGCCGUCGUGTT-3′

In summary, vascular injury suppresses the expression of SRF and Myocardin, resulting in decreased expression of SMC-specific marker genes, which is characterized by enhanced proliferation and migration, and eventually leads to vascular hyperplasia. The treatment of SMC with Andrographolide activates the endothelin signaling pathway and promotes the interaction of EDNA and EDNB with the Myocardin-SRF complex to induce SMC-specific marker gene expression (Figure 7H).

## Discussion

This study provides evidence that Andrographolide regulates pathological vascular remodeling. The treatment of rat SMCs with Andrographolide activates the endothelin signaling pathway and promotes interaction of EDNA and EDNB with the Myocardin-SRF complex to induce SMC-specific marker gene expression, resulting in restrained pathological vascular remodeling. We provide first evidence that Traditional Chinese Medicine Andrographolide regulates pathological vascular remodeling through interaction between endothelin receptors with the Myocardin-SRF complex.

Endothelin was first identified from bovine endothelial cell in 1985 (35). The endothelin/sarafotoxin family consists of three isoforms, including ET-1, ET-2, and ET-3 (14). The expression of ET-1 is much higher than ET-2 and ET-3 (Supplementary Figure 5A). The transcription level of ET-1 was obviously changed after Andrographolide treatment. We focused on ET-1 in this study and evaluated whether and how Andrographolide regulates pathological vascular remodeling. However, as a secreted peptide, it is difficult to evaluate the concentration and activation of ET in tissues and organs. We monitored the transcription level of ET-1 in SMCs by real-time PCR following Andrographolide treatment.

ET-1 interacts with cognate receptors to regulate vasoconstriction. Three types of G protein-coupled endothelin/sarafotoxin family receptors have been identified, including EDNRA, EDNRB, and EDNRB_2_ (36). Both ET-1 and ET-2 can activate these three kinds of receptors, whereas ET-3 only activates EDNRB and EDNRB_2_. EDNRB is a major receptor that is expressed in SMCs (Supplementary Figure 5B). However, Andrographolide treatment dramatically enhanced the expression of EDNRA and EDNRB. In this study, we determined whether Andrographolide treatment regulates pathological vascular remodeling by the interaction between ET-1 and EDNRA or EDNRB.

In this study, we identified a novel mechanism where Andrographolide activates the endothelin signaling pathway and promotes the interaction of receptors EDNA and EDNB with the Myocardin-SRF complex to induce SMC-specific marker gene expression. Previous studies demonstrated that ET-1 promotes proliferation and migration of vascular smooth muscle cells. Although the majority of ET-1 is generated by endothelial cells, ET-1 can also be released by vascular SMCs (37, 38). However, whether and how Andrographolide regulates SMC differentiation is not well defined. Identification of SRF and Myocardin represents tremendous progress in defining SMC phenotypic switching. SRF dimerism binds to the CArG element that exists in the promoter regions of multiple SMC marker genes. Myocardin induces multiple SMC marker gene expression by binding to SRF. In this study, we demonstrated that Andrographolide promoted the interaction of EDNA and EDNB with Myocardin-SRF, and induced CArG boxes containing SMC-specific marker gene expression (Figure 7G).

Pathological vascular remodeling involves SMC proliferation, endothelial cell inflammation, collagen synthesis (39), and macrophages, etc. We performed a CCK8 cell proliferation assay and BRDU cell incorporation assay to evaluate whether Andrographolide suppresses SMC proliferation. We also performed a Boyden chamber migration assay and spheroid sprouting assay to determine whether Andrographolide inhibits SMC migration. Furthermore, we defined that Andrographolide activates the endothelin signaling pathway and promotes the interaction of receptor EDNA and EDNB with Myocardin-SRF to induce CArG boxes containing SMC-specific marker gene expression. The data are very interesting. However, these data cannot account for every detail that happens during pathological vascular remodeling. More studies need to focus on how Andrographolide regulates endothelial cell behaviors during pathological vascular remodeling.

Although we provide evidence that Andrographolide regulates pathological vascular remodeling by inducing the interaction of EDNRA and EDNRB with the Myocardin-SRF complex, resulting in enhanced expression of CArG boxes containing SMC-specific marker genes. Some other signaling pathways may also be involved during pathological vascular remodeling. Our real-time PCR data exhibited that IL-15, IL-18, IGF1, and Hif1a were also regulated in SMCs after Andrographolide treatment (Supplementary Figure 4).

Extra matric deposition, degradation, and rearrangement are critical for development of the vascular system and aging of tissue and organs. Our real-time PCR data indicated that expression of versicon, collagen I, collagen II, and fibronectin were decreased in SMCs following Andrographolide treatment (Supplementary Figure 10).

In summary, this study not only demonstrates the critical role of Andrographolide on regulating pathological vascular remodeling, but also identifies a novel mechanism where Andrographolide activates the endothelin signaling pathway and promotes the interaction of EDNA and EDNB with Myocardin-SRF to induce CArG boxes containing SMC-specific marker gene expression.

## Data Availability Statement

The original contributions presented in the study are included in the article/Supplementary Material, further inquiries can be directed to the corresponding author/s.

## Ethics Statement

The animal study was reviewed and approved by Experimental Animal Ethics Committee of Chengdu University of Traditional Chinese Medicine.

## Author Contributions

YW, LY, HX, and LS designed the research. WH, XW, ZJ, ZW, QG, ZC, SZ, HZ, and LZ performed the experiments. JH, LZ, XZ, and WH analyzed the data. YW and WH wrote and revised the manuscript. All authors contributed to the article and approved the submitted version.

## Funding

This study was supported by Grants (81870363 and 81741007) from the National Natural Science Foundation of China, Grant (2020JDTD0025) from the Science and Technology Departments of Sichuan Province, and Grants (008066, 030038199, 030041023, 030041224, 0300500092, 0300510026, 030055180, 319020056, 242030016, and MPRC2021038) from Cheng Du University of Traditional Chinese Medicine.

## Conflict of Interest

The authors declare that the research was conducted in the absence of any commercial or financial relationships that could be construed as a potential conflict of interest.

## Publisher's Note

All claims expressed in this article are solely those of the authors and do not necessarily represent those of their affiliated organizations, or those of the publisher, the editors and the reviewers. Any product that may be evaluated in this article, or claim that may be made by its manufacturer, is not guaranteed or endorsed by the publisher.

## References

[1] Alexander MR Owens GK. Epigenetic control of smooth muscle cell differentiation and phenotypic switching in vascular development and disease. Annu Rev Physiol. (2012) 74:13–40. 10.1146/annurev-physiol-012110-142315 22017177

[2] Harika S Arti V Sandeep A Daniel A Oge A Fang L. Pharmacological inhibition of β-catenin prevents EndMT *in vitro* and vascular remodeling *in vivo* resulting from endothelial Akt1 suppression. Biochem Pharmacol. (2019) 164:205–15. 10.1016/j.bcp.2019.04.016 30991049 PMC6525030

[3] Chiang HY Korshunov VA Serour A Shi F Sottile J. Fibronectin is an important regulator of flow-induced vascular remodeling. Arterioscler Thromb Vasc Biol. (2009) 29:1074–9. 10.1161/ATVBAHA.108.181081 19407246 PMC3091823

[4] Penn DL Witte SR Komotar RJ Sander Connolly E Jr. The role of vascular remodeling and inflammation in the pathogenesis of intracranial aneurysms. J Clin Neurosci. (2014) 21:28–32. 10.1016/j.jocn.2013.07.004 24120708

[5] Chan S Yan C. PDE1 isozymes, key regulators of pathological vascular remodeling. Curr Opin Pharmacol. (2011) 11:720–4. 10.1016/j.coph.2011.09.002 21962439 PMC3225597

[6] Cai Y Knight WE Guo S Li JD Knight PA Yan C. Vinpocetine suppresses pathological vascular remodeling by inhibiting vascular smooth muscle cell proliferation and migration. J Pharmacol Exp Ther. (2012) 343:479–88. 10.1124/jpet.112.195446 22915768 PMC3477207

[7] Nagao M Lyu Q Zhao Q Wirka RC Bagga J Nguyen T. Coronary disease-associated gene TCF21 inhibits smooth muscle cell differentiation by blocking the myocardin-serum response factor pathway. Circ Res. (2020) 126:517–29. 10.1161/CIRCRESAHA.119.315968 31815603 PMC7274203

[8] McDonald OG Wamhoff BR Hoofnagle MH Owens GK. Control of SRF binding to CArG box chromatin regulates smooth muscle gene expression *in vivo*. J Clin Invest. (2006) 116:36–48. 10.1172/JCI26505 16395403 PMC1323266

[9] Wang Z Wang DZ Hockemeyer D McAnally J Nordheim A Olson EN. Myocardin and ternary complex factors compete for SRF to control smooth muscle gene expression. Nature. (2004) 428:185–9. 10.1038/nature02382 15014501

[10] Yoshida T Hoofnagle MH Owens GK. Myocardin and Prx1 contribute to angiotensin II-induced expression of smooth muscle α-actin. Circul Res. 94:1075–82. 10.1161/01.RES.0000125622.46280.95 15016729

[11] Liao XH Wang N Zhao DW Zheng DL Zheng L Xing WJ. STAT3 protein regulates vascular smooth muscle cell phenotypic switch by interaction with myocardin. J Biol Chem. (2015) 290:19641–52. 10.1074/jbc.M114.630111 26100622 PMC4528129

[12] Davis-Dusenbery BN Chan MC Reno KE Weisman AS Layne MD Lagna G. A down-regulation of Krüppel-like factor-4 (KLF4) by microRNA-143/145 is critical for modulation of vascular smooth muscle cell phenotype by transforming growth factor-β and bone morphogenetic protein 4. J Biol Chem. (2011) 286:28097–110. 10.1074/jbc.M111.236950 21673106 PMC3151055

[13] Rahman NT Schulz VP Wang L Gallagher PG Denisenko O Gualdrini F. augments megakaryocyte maturation by enhancing the SRF regulatory axis. Blood Adv. (2018) 2:2691–703. 10.1182/bloodadvances.2018019448 30337297 PMC6199649

[14] Dhaun N Webb DJ. Endothelins in cardiovascular biology and therapeutics. Nat Rev Cardiol. (2019) 16:491–502. 10.1038/s41569-019-0176-3 30867577

[15] Luscher TF Barton M. Endothelins and endothelin receptor antagonists: therapeutic considerations for a novel class of cardiovascular drugs. Circulation. (2000) 102:2434–40. 10.1161/01.cir.102.19.2434 11067800

[16] Kawanabe Y Nauli SM. Endothelin. Cell Mol Life Sci. (2011) 2:195–203. 10.1007/s00018-010-0518-0 20848158 PMC3141212

[17] Miyauchi T Sakai S. Endothelin and the heart in health and diseases. Peptides. (2019) 111:77–88. 10.1016/j.peptides.2018.10.002 30352269

[18] Agapitov AV Haynes WG. Role of endothelin in cardiovascular disease. J Renin Angiotensin Aldosterone Syst. (2002) 3:1–15. 10.3317/jraas.2002.001 11984741

[19] Planas-Rigol E Terrades-Garcia N Corbera-Bellalta M Lozano E Alba MA Segarra M. Endothelin-1 promotes vascular smooth muscle cell migration across the artery wall: a mechanism contributing to vascular remodelling and intimal hyperplasia in giant-cell arteritis. Ann Rheum Dis. (2017) 9:1624–34. 10.1136/annrheumdis-2016-210792 28606962

[20] Takahashi M. The role of endothelin-1 in vascular remodeling *in vivo*. Cardiovasc Res. (2006) 1:4–5. 10.1016/j.cardiores.2006.05.006 16730685

[21] Wu Z Xu H Xu Y Fan W Yao H Wang Y. Andrographolide promotes skeletal muscle regeneration after acute injury through epigenetic modulation. Eur J Pharmacol. (2020) 888:173470. 10.1016/j.ejphar.2020.173470 32822641

[22] Peng YL Wang Y Tang N Sun DD Lan YL Yu ZL Zhao XY. Andrographolide inhibits breast cancer through suppressing COX-2 expression and angiogenesis via inactivation of p300 signaling and VEGF pathway. J Exp Clin Canc Res. (2018) 37:1–14. 10.1186/s13046-018-0926-9 30314513 PMC6186120

[23] Shen KK Ji LL Lu B Xu C Gong CY Morahan G Wang ZT. Andrographolide inhibits tumor angiogenesis via blocking VEGFA/VEGFR2-MAPKs signaling cascade. Chem-Biol Interact. (2014) 218:99–106. 10.1016/j.cbi.2014.04.020 24814888

[24] Kajal K Panda AK Bhat J Chakraborty D Bose S Bhattacharjee P. Andrographolide binds to ATP-binding pocket of VEGFR2 to impede VEGFA-mediated tumor-angiogenesis. Sci Rep. (2019) 9:1–10. 10.1038/s41598-019-40626-2 30858542 PMC6412047

[25] Dai JW Lin YY Duan YF Li ZX Zhou DL Chen WS. Andrographolide inhibits angiogenesis by inhibiting the Mir-21-5p/TIMP3 signaling pathway. Int J Biol Sci. (2017) 5:660–8. 10.7150/ijbs.19194 28539838 PMC5441182

[26] Wang YJ Wang JT Fan QX Geng JG. Andrographolide inhibits NF-kappaBeta activation and attenuates neointimal hyperplasia in arterial restenosis. Cell Res. (2007) 17:933–41. 10.1038/cr.2007.89 17943075

[27] Yao H Wu ZQ Xu YM Xu H Lou GH Jiang Q. Andrographolide attenuates imbalance of gastric vascular homeostasis induced by ethanol through glycolysis pathway. Sci Rep. (2019) 9:1–10. 10.1038/s41598-019-41417-5 30899067 PMC6428857

[28] Kumar A Lindner V. Remodeling with neointima formation in the mouse carotid artery after cessation of blood flow. Arterioscler Thromb Vasc Biol. (1997) 10:2238–44. 10.1161/01.atv.17.10.2238 9351395

[29] Wang Y Xu YM Yan SY Cao KX Zeng XQ Zhou YQ. Adenosine kinase is critical for neointima formation after vascular injury by inducing aberrant DNA hypermethylation. Cardiovasc Res. (2021) 117:561–75. 10.1093/cvr/cvaa040 32065618 PMC7820850

[30] Wang XB Hu GQ Gao XW Wang Y Zhang W Harmon EY. The induction of yes-associated protein expression after arterial injury is crucial for smooth muscle phenotypic modulation and neointima formation. Arterioscl Throm Vasc. (2012) 32:2662–9. 10.1161/Atvbaha.112.254730 22922963 PMC3475752

[31] Xu S Fu J Chen J Xiao P Lan T Le K. Development of an optimized protocol for primary culture of smooth muscle cells from rat thoracic aortas. Cytotechnology. (2009) 61:65–72. 10.1007/s10616-009-9236-6 19898948 PMC2795140

[32] Zhu N Xiang YJ Zhao XY Cai CH Chen H Jiang WB. Thymoquinone suppresses platelet-derived growth factor-BB-induced vascular smooth muscle cell proliferation, migration and neointimal formation. J Cell Mol Med. (2019) 12:8482–92. 10.1111/jcmm.14738 31638340 PMC6850929

[33] Schmitt BM Boewe AS Becker V Nalbach L Gu Y Gotz C. Protein kinase CK2 regulates nerve/glial antigen (NG)2-mediated angiogenic activity of human pericytes. Cells. (2020) 9:9061546. 10.3390/cells9061546 32630438 PMC7348826

[34] Wang Y Hu GQ Liu F Wang XB Wu MF Schwarz JJ. Deletion of yes-associated protein (YAP) specifically in cardiac and vascular smooth muscle cells reveals a crucial role for YAP in mouse cardiovascular development. Circul Res. (2014) 114:957–65. 10.1161/Circresaha.114.303411 24478334 PMC4049286

[35] Hickey KA Rubanyi G Paul RJ Highsmith RF. Characterization of a coronary vasoconstrictor produced by cultured endothelial cells. Am J Physiol. (1985) 248:C550–6. 10.1152/ajpcell.1985.248.5.C550 3993773

[36] Rodriguez-Pascual F Busnadiego O Lagares D Lamas S. Role of endothelin in the cardiovascular system. Pharmacol Res. (2011) 63:463–72. 10.1016/j.phrs.2011.01.014 21296157

[37] Tian XY Zhang QY Huang YQ Chen SL Tang CS Sun Y. Endothelin-1 downregulates sulfur dioxide/aspartate aminotransferase pathway via reactive oxygen species to promote the proliferation and migration of vascular smooth muscle cells. Oxid Med Cell Longev. (2020) 2020:9367673. 10.1155/2020/9367673 32089786 PMC7008293

[38] Wort SJ Woods M Warner TD Evans TW Mitchell JA. Endogenously released endothelin-1 from human pulmonary artery smooth muscle promotes cellular proliferation: relevance to pathogenesis of pulmonary hypertension and vascular remodeling. Am J Respir Cell Mol Biol. (2001) 25:104–10. 10.1165/ajrcmb.25.1.4331 11472982

[39] Liu X Zhang S Wang X Wang Y Song J Sun C. Endothelial cell-derived SO(2) controls endothelial cell inflammation, smooth muscle cell proliferation, and collagen synthesis to inhibit hypoxic pulmonary vascular remodelling. Oxid Med Cell Longev. (2021) 2021:5577634. 10.1155/2021/5577634 33953829 PMC8068783

