# Supplementary material for: Andrographolide Promotes Interaction Between Endothelin-Dependent EDNRA/EDNRB and Myocardin-SRF to Regulate Pathological Vascular Remodeling
Source: Front Cardiovasc Med. 2022 Jan 20;8:783872. doi: 10.3389/fcvm.2021.783872 (PMC8810813; doi:10.3389/fcvm.2021.783872)
Supplement: Supplementary file 1 [file Data_Sheet_1.docx]

**Supplementary materials**

**Andrographolide promotes interaction between endothelin-dependent EDNR1/EDNRB and Myocardin-SRF to regulate pathological vascular remodeling**

Wangming Hu ^1†^, Wu Xiao ^1†^, Zhong Jin ^1†^, Zheng Wang ^1^, Qiru Guo ^1^, Zixian Chen ^2^, Song Zhu ^3^, Haidi Zhang ^3^, Huo Jian ^4^, Lingling Zhang ^1^, Xin Zhou ^1^, Lan Yang ^1^, Huan Xu ^1^, Liangqing Shi ^1^, Yong Wang ^1#^

^1^ College of Basic Medicine, Chengdu University of Traditional Chinese Medicine, Chengdu, China.

^2^ School of Ethnic Medicine, Chengdu University of Traditional Chinese Medicine, Chengdu, China.

^3^ Chengdu University of Traditional Chinese Medicine, Hospital of Chengdu University of Traditional Chinese Medicine, Chengdu, China.

^4^ Chengdu Women's and Children's Central Hospital, School of Medicine, University of Electronic Science and Technology of China, Chengdu, China.

^†^ These authors have contributed equally to this work and share first authorship

^#^Address for Correspondence:

Yong Wang, Ph.D

Basic Medical College, Cheng Du University of Traditional Chinese Medicine, Chengdu, China

Email: [yongwang1008@hotmail.com](mailto:yongwang1008@hotmail.com) (wangyong@cdutcm.edu.cn)

**Supplementary Figure 1**. **Andrographolide treatment did not change media smooth muscle layer area in carotid artery ligation model**. (A) Andrographolide (10mg/kg) was administered by intraperitoneal injection for 14 consecutive days after left common carotid artery ligation. H&E staining performed to visualized vascular morphological changes. The media layer area of the Andrographolide group and the vehicle group was measured using Image J software (n=5). (B) Quantification of media smooth muscle layer area after 21 consecutive days treatment with Andrographolide (n=5). Data represented as means ± SEM. * P < 0.05.

**Supplementary Figure 2.** **Andrographolide is critical in regulating smooth muscle cells phenotypic switching.** (A). Rat SMCs treated with Andrographolide (5μM) for 30 hours, real time PCR performed to detect mRNA levels of SMC differentiated genes (n=6). (B). Proliferation of rat SMCs were induced by PDGF-BB treatment (25ng/ml). Real time PCR performed to determine expression of SMC proliferation related genes (n=6). (C). Differentiation of SMCs were induced by rapamycin treatment (100nM/L). Real time PCR performed to determine expression of SMC differentiation related genes (n=6). (D). Differentiation of rat SMCs were mimicked by starvation (0.2%FBS), following Andrographolide (5μM) treatment. Real time PCR was performed to determine SMCs specific marker genes expression (n=6). Data are expressed as mean ± SEM. * P <0.05.

**Supplementary Figure 3. Andrographolide treatment did not change expression of proliferation markers genes PCNA and Ki67 in media smooth muscle layer.** (A) Immunohistochemical staining against proliferation markers PCNA in media smooth muscle layer. The number of PCNA positive SMCs were quantified (n=5). (B) Immunohistochemical staining of the proliferation markers Ki67 and analyze the number of Ki67 positive cells in media layer (n=5). Data represented as mean ± SEM. * P < 0.05.

**Supplementary Figure 4. Multiple signaling pathways in SMC were Screened after Andrographolide treatment.** Rat SMCs were treated with Andrographolide (5μM) for 30 hours, and multiple signaling pathways were determined by Real time PCR (n=6). Data presented as mean ± SEM. * P < 0.05.

**Supplementary Figure 5.** **The expression of endothelins and receptors in SMCs.**

(A) Total RNA from rat SMCs were extracted using Trizol reagent. The expression of ET1, ET2 and ET3 mRNA level were evaluated by real time PCR, and EDNRA, EDNRB mRNA level exhibited in (B) (n=6). Data represented as mean ± SEM. * P < 0.05.

**Supplementary Figure 6.** **Andrographolide activates Endothelin family.** (A). Rat SMCs treated with Andrographolide (1μM) for 30h, expression of ET1, ET2, ET3 mRNA level were determined by real time PCR, and EDNRA, EDNRB mRNA level exhibited in (B) (n=6). (C). Proliferation of rat SMCs were induced by PDGF-BB (25ng/ml) treatment, following Andrographolide (5μM) treatment. The expression of ET1, ET2, ET3 mRNA level were determined by real time PCR. EDNRA, EDNRB mRNA level (D) that detected by real time PCR exhibited in (D) (n=6). Data presented as mean ± SEM. * P < 0.05.

**Supplementary Figure 7. Andrographolide promotes expression of** **SRF and Myocardin**. (A) Treated rat SMCs with Andrographolide (1μM) for 30 hours, and the mRNA transcription level of SRF and Myocardin was detected by real time PCR (n=6). (B). Proliferation of rat SMCs were treated with PDGF-BB (25ng/ml), following Andrographolide (5μM) treatment, the expression of SRF and Myocardin mRNA level was evaluated by real time PCR (n=6). Data presented as mean ± SEM. * P < 0.05.

**Supplementary Figure 8.** **Inhibition of EDNRA and EDNRB promotes rat SMC proliferation.** (A). Rat SMCs were treated with Macitentan (1μM) overnight, following incubated with BRDU labeling buffer for 20 hours. Immunofluorescence staining performed to observe BRDU incorporation, BRDU positive cells were quantified in (B). (C). Treated rat SMCs with Macitentan (1μM) for 24 hours, the viability of SMCs was detected by CCK8 cell proliferation assay (n=8). Data presented as mean ± SEM. * P < 0.05.

**Supplementary Figure 9.** **Inhibition of EDNRA and EDNRB promotes rat SMC migration.** (A). Spheroid sprouting assay performed to determine rat SMCs migration after Macitentan (1μM) treatment for 12 hours. The sprouting and sprouting length were quantified in (B) and (C) (n = 8). Data presented as mean ± SEM. * P < 0.05.

**Supplementary Figure 10.** **Inhibition of EDNRA and EDNRB promotes rat SMC proliferation.** Rat SMCs were treated with Macitentan (1μM), Andrographolide (5μM) for 24h, the cell viability is detected by CCK8 (n=8). The analysis data are expressed as means ± SEM. *P<0.05.

**Supplementary Figure 11. Deletion EDNRA in rat SMC by siRNA targeting EDNRA.** Rat SMCs were transfected with si-control or siRNA targeting EDNRA, real time PCR was used to validate EDNRA deletion efficiency after transfection at 30 h (A) and 48 h (B). siRNA sequence was chosen from three different sequences. Data presented as mean ± SEM. * P < 0.05.

**Supplementary Figure 12. Deletion SRF in rat SMC by siRNA targeting SRF.** Rat SMCs were transfected with si-control or siRNA targeting SRF, real time PCR was used to validate SRF deletion efficiency after transfection at 30 h (A) and 48 h (B). siRNA sequence was chosen from three different sequences. Data presented as mean ± SEM. * P < 0.05.

**Supplementary Figure 13.** **Andrographolide suppressed differentiation of rat SMCs after EDNRA gene silencing.** Rat SMCs were transfected with small interfering si-EDNRA for 4 hours, and then treated with Andrographolide (5μM) for 30 hours, the mRNA level of SMCs specific marker genes, including SRF, Myocardin, MHC, calponin, SM22α, smooth muscle α-actin, and proliferation-related genes, including PCNA, were detected by real time PCR (n=6). Data presented as mean ± SEM. * P < 0.05.

**Supplementary Figure 14.** **Andrographolide** **enhances the differentiation of rat SMCs after SRF gene silencing.** Rat SMCs were transfected with small interfering RNA si-SRF for 4 hours, and then treated with Andrographolide (5μM) for 30 hours, the mRNA level of SMCs specific marker genes, including SRF, Myocardin, MHC, calponin, SM22α, smooth muscle α-actin, KLF4, MRTFA and proliferation-related genes including CDKN1A, CDKN1B, PCNA were detected by real time PCR (n=6). Data presented as mean ± SEM. * P < 0.05.

**Supplementary Figure 15.** **Andrographolide attenuates deposition of extracellular matrix.** Rat SMCs were treated with Andrographolide (5μM) for 30 hours, then the mRNA levels of Versicon, Collagen I, Collagen II, Has3 and Fibronectin were detected by real time PCR (n=6). Data presented as mean ± SEM. * P < 0.05.
