# Supplementary material for: Andrographolide Promotes Interaction Between Endothelin-Dependent EDNRA/EDNRB and Myocardin-SRF to Regulate Pathological Vascular Remodeling
Source: Front Cardiovasc Med. 2022 Jan 20;8:783872. doi: 10.3389/fcvm.2021.783872 (PMC8810813; doi:10.3389/fcvm.2021.783872)
Supplement: Supplementary file 2 [file Data_Sheet_2.PDF]

S Figure 1

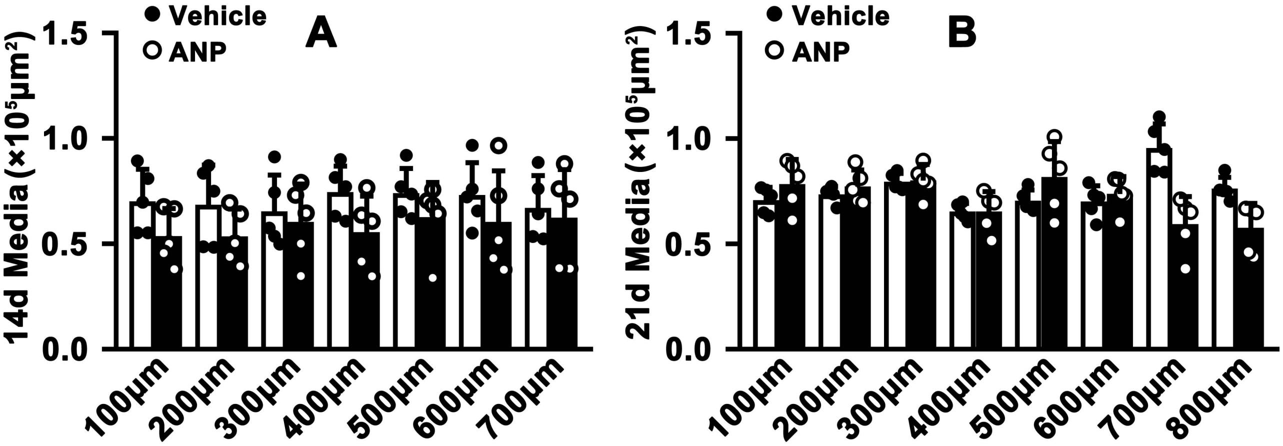

Vehicle
ANP
A
14d Media (×10⁵µm²)
1.5
1.0
0.5
0.0
100µm
200µm
300µm
400µm
500µm
600µm
700µm
Vehicle
ANP
B
21d Media (×10⁵µm²)
1.5
1.0
0.5
0.0
100µm
200µm
300µm
400µm
500µm
600µm
700µm
800µm

S Figure 2

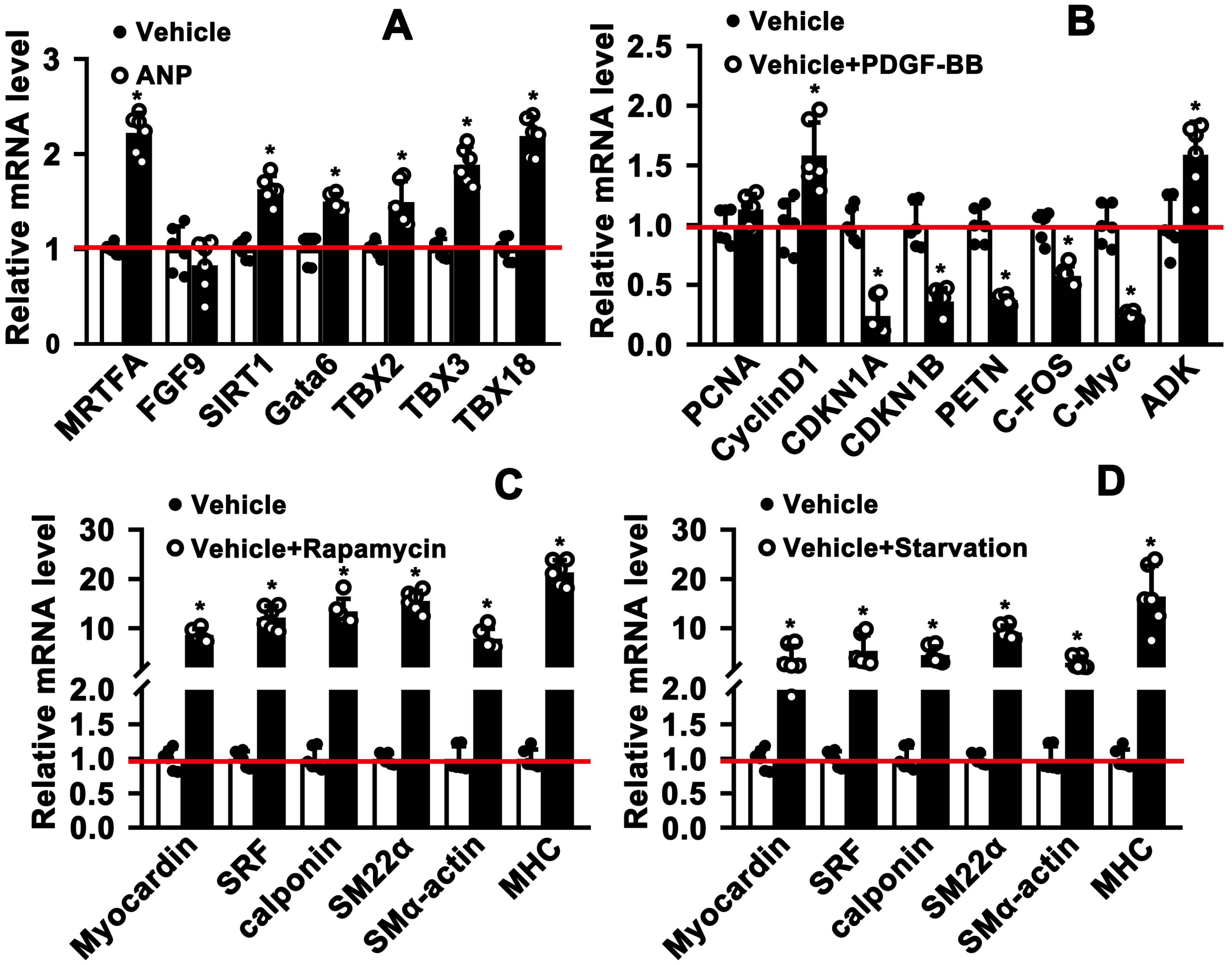

A
Vehicle
ANP
Relative mRNA level
MRTFA
FGF9
SIRT1
Gata6
TBX2
TBX3
TBX18
B
Vehicle
Vehicle+PDGF-BB
PCNA
CyclinD1
CDKN1A
CDKN1B
PETN
C-FOS
C-Myc
ADK
C
Vehicle
Vehicle+Rapamycin
Myocardin
SRF
calponin
SM22α
SMα-actin
MHC
D
Vehicle
Vehicle+Starvation

S Figure 3

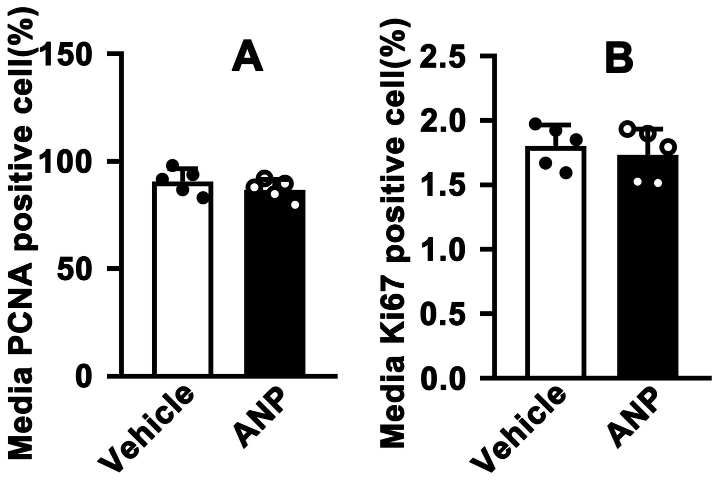

A
Media PCNA positive cell(%)
150
100
50
0
Vehicle
ANP
B
Media Ki67 positive cell(%)
2.5
2.0
1.5
1.0
0.5
0.0
Vehicle
ANP

S Figure 4

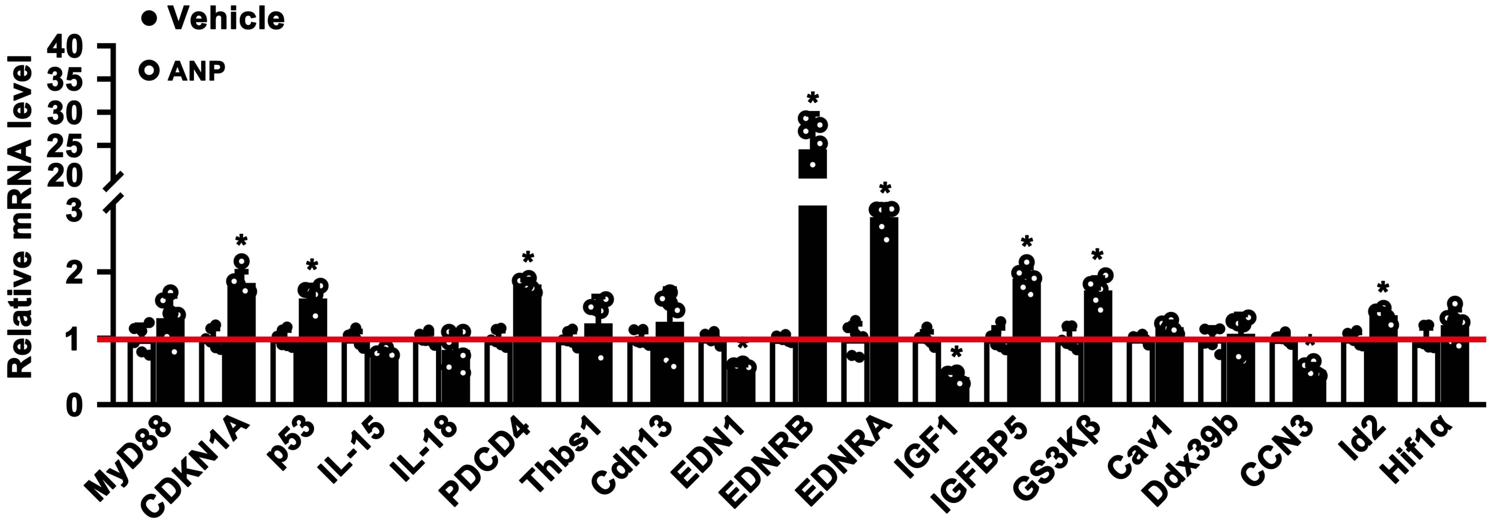

Vehicle
ANP
Relative mRNA level
40
35
30
25
20
3
2
1
0
*
MyD88
CDKN1A
p53
IL-15
IL-18
PDCD4
Thbs1
Cdh13
EDN1
EDNRB
EDNRA
IGF1
IGFBP5
GS3Kβ
Cav1
Ddx39b
CCN3
Id2
Hif1α

S Figure 5

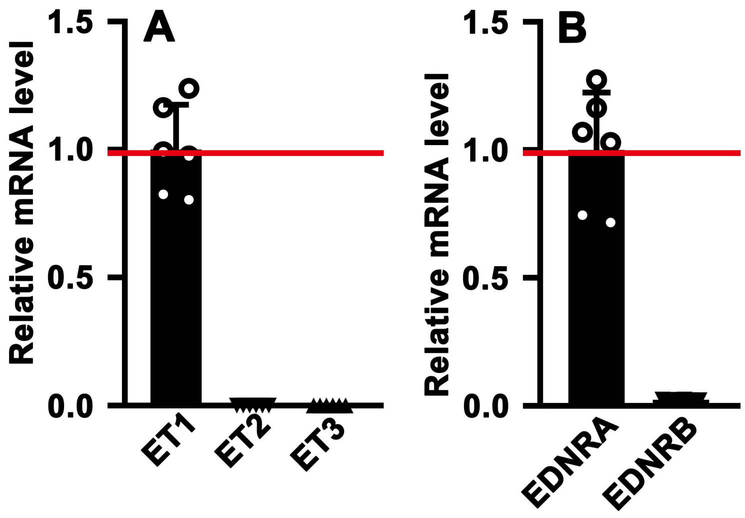

S Figure 6

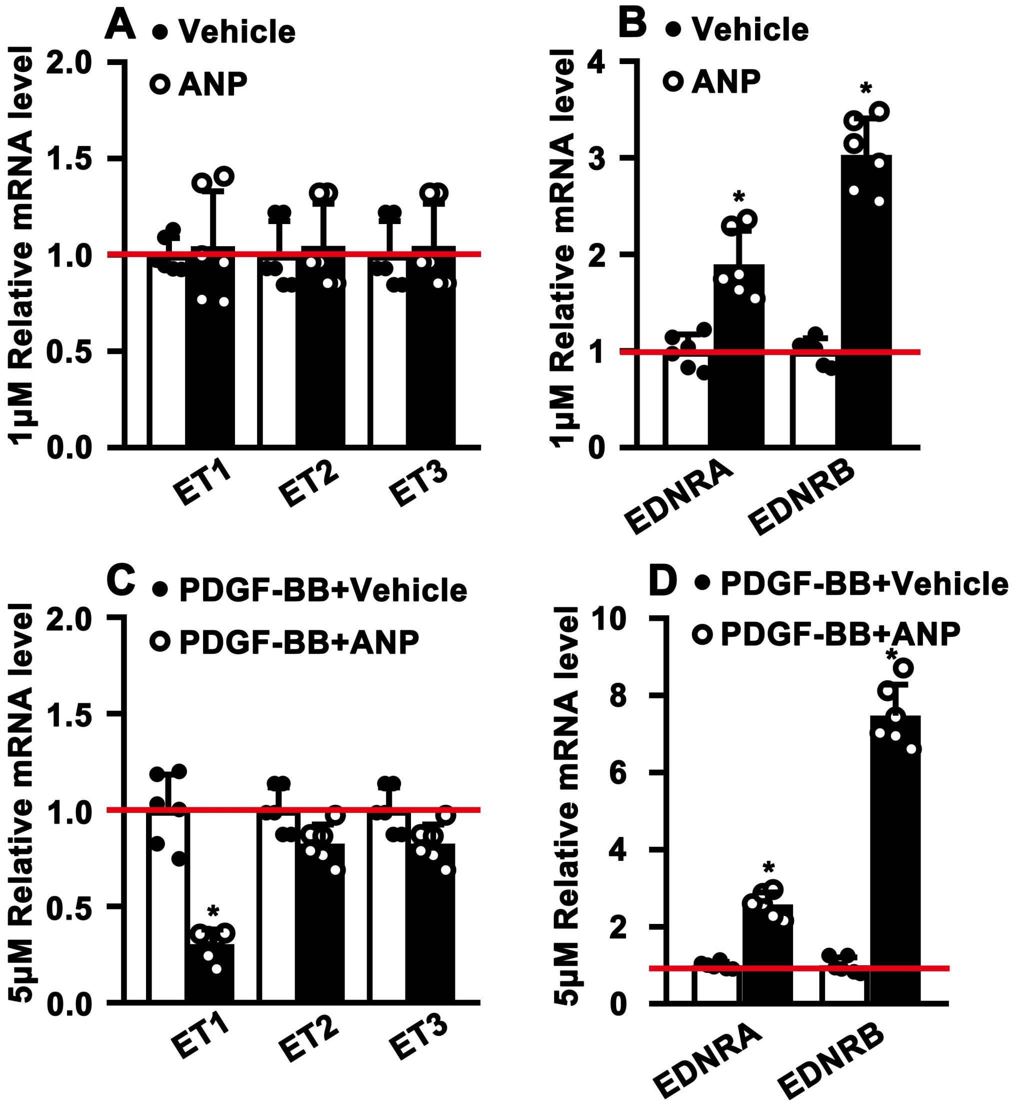

A
Vehicle
ANP
1μM Relative mRNA level
2.0
1.5
1.0
0.5
0.0
ET1
ET2
ET3
B
Vehicle
ANP
1μM Relative mRNA level
4
3
2
1
0
*
*
EDNRA
EDNRB
C
PDGF-BB+Vehicle
PDGF-BB+ANP
5μM Relative mRNA level
2.0
1.5
1.0
0.5
0.0
*
ET1
ET2
ET3
D
PDGF-BB+Vehicle
PDGF-BB+ANP
5μM Relative mRNA level
10
8
6
4
2
0
*
*
EDNRA
EDNRB

S Figure 7

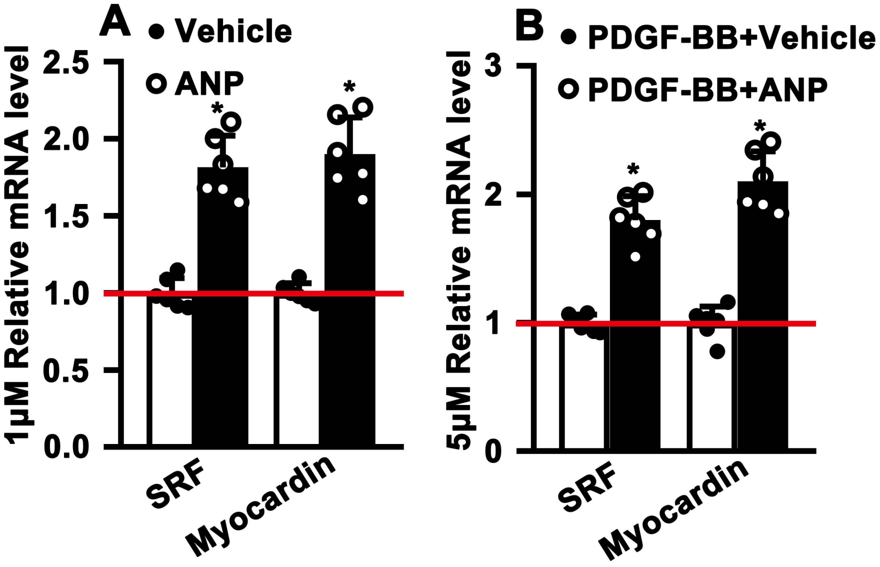

A
Vehicle
ANP
1μM Relative mRNA level
2.5
2.0
1.5
1.0
0.5
0.0
SRF
Myocardin
B
PDGF-BB+Vehicle
PDGF-BB+ANP
5μM Relative mRNA level
3
2
1
0
SRF
Myocardin

S figure 8

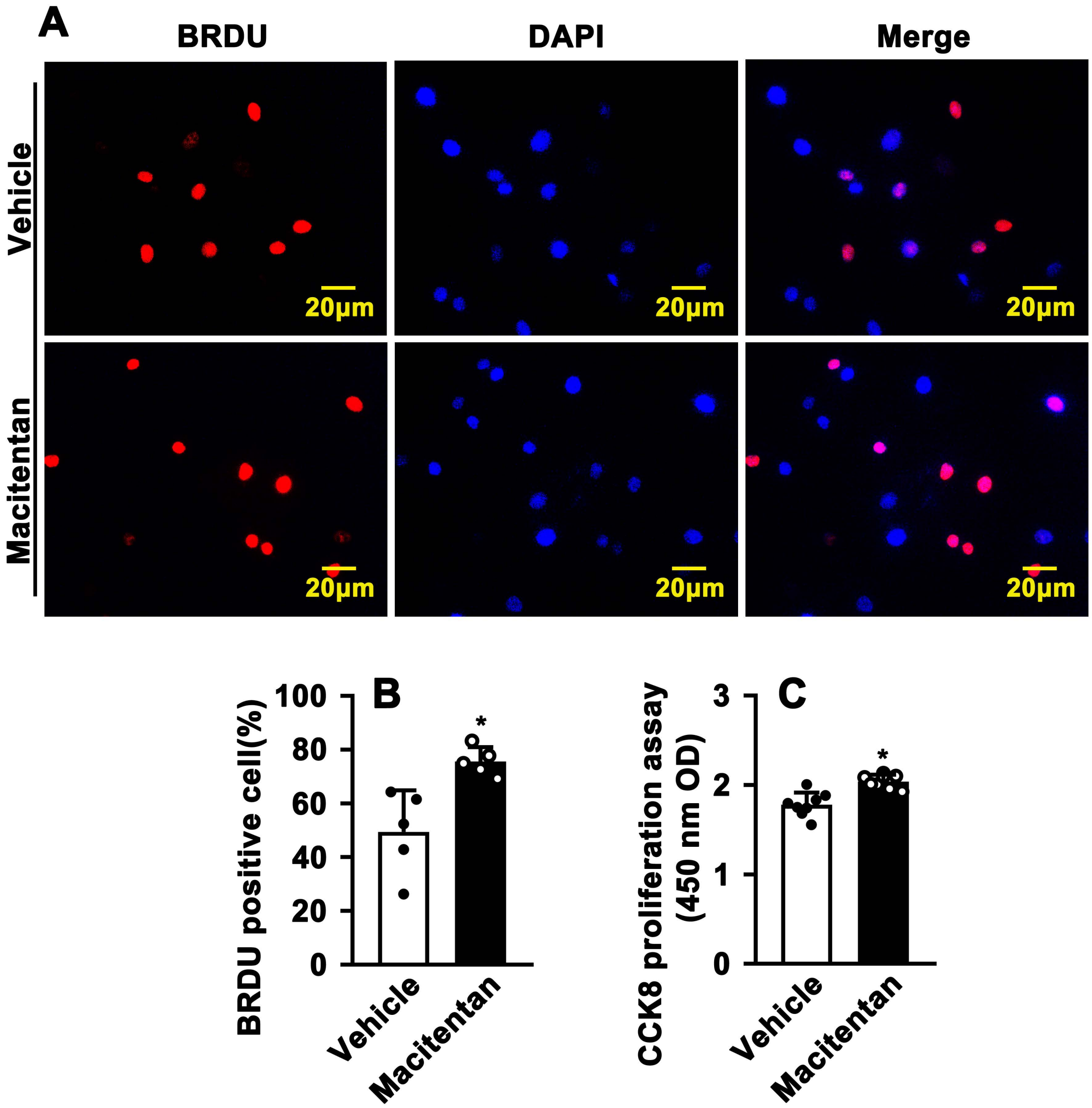
A
BRDU
DAPI
Merge
Vehicle
Macitentan
20μm
B
BRDU positive cell(%)
100
80
60
40
20
0
*
Vehicle
Macitentan
C
CCK8 proliferation assay (450 nm OD)
3
2
1
0
*
Vehicle
Macitentan

S figure 9

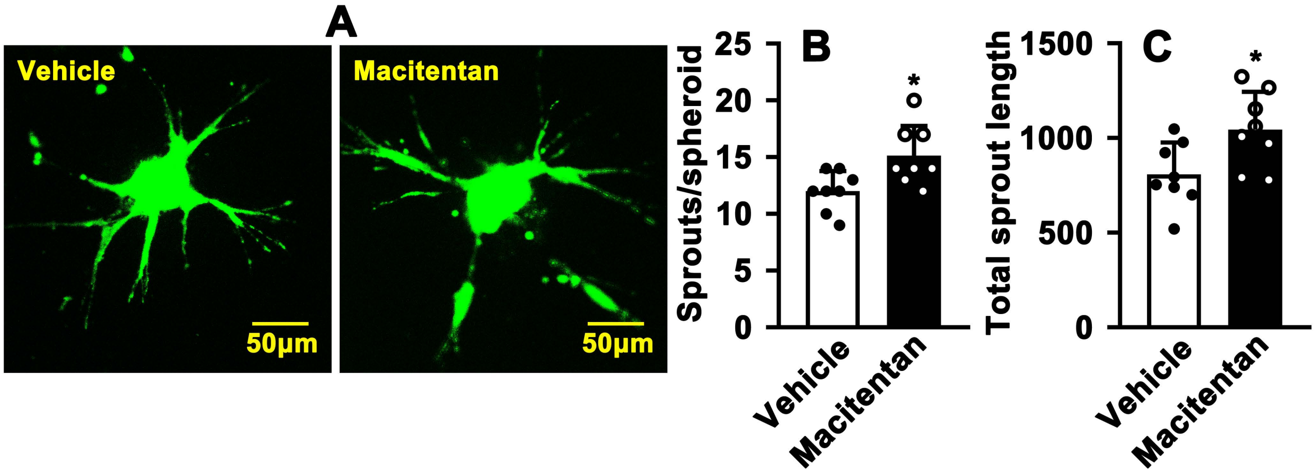

A
Vehicle
50μm
Macitentan
50μm
B
Sprouts/spheroid
25
20
15
10
5
0
Vehicle
Macitentan
*
C
Total sprout length
1500
1000
500
0
Vehicle
Macitentan
*

S Figure 10

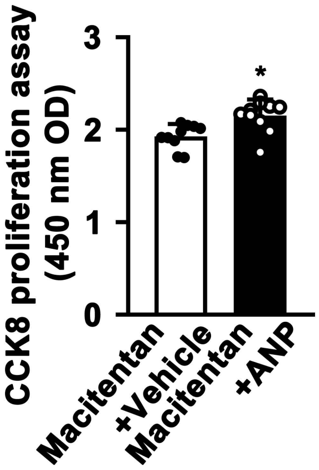

CCK8 proliferation assay
(450 nm OD)
3
2
1
0
*
Macitentan
+Vehicle
Macitentan
+ANP

S Figure 11

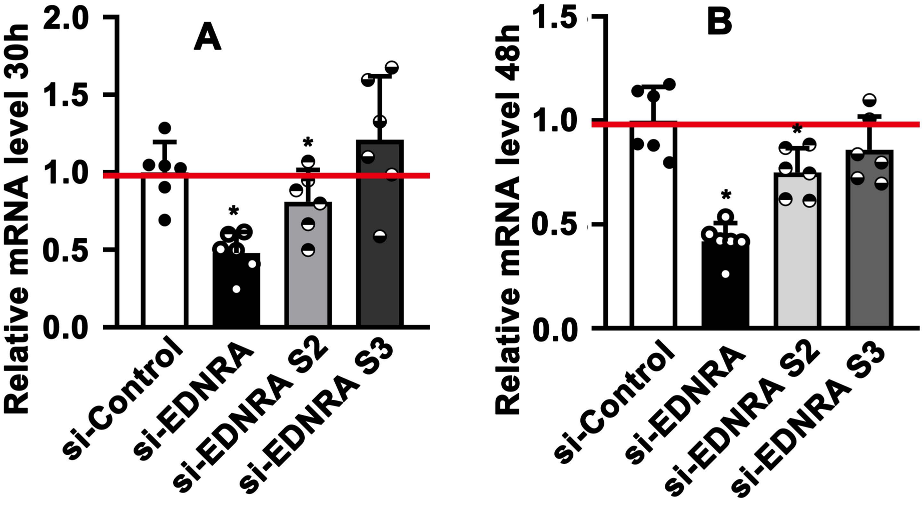
A
Relative mRNA level 30h
2.0
1.5
1.0
0.5
0.0
*
*
si-Control
si-EDNRA
si-EDNRA S2
si-EDNRA S3
B
Relative mRNA level 48h
1.5
1.0
0.5
0.0
*
*
si-Control
si-EDNRA
si-EDNRA S2
si-EDNRA S3

S Figure 12

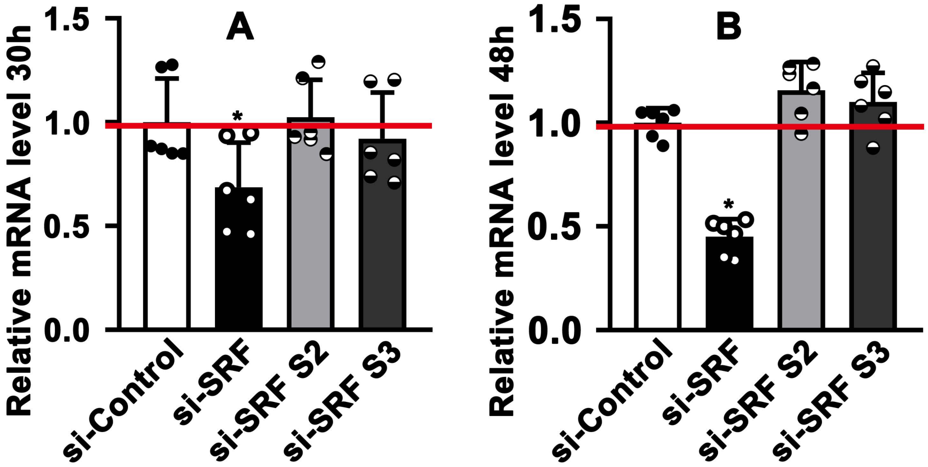

A
Relative mRNA level 30h
1.5
1.0
0.5
0.0
*
si-Control
si-SRF
si-SRF S2
si-SRF S3
B
Relative mRNA level 48h
1.5
1.0
0.5
0.0
*
si-Control
si-SRF
si-SRF S2
si-SRF S3

S Figure 13

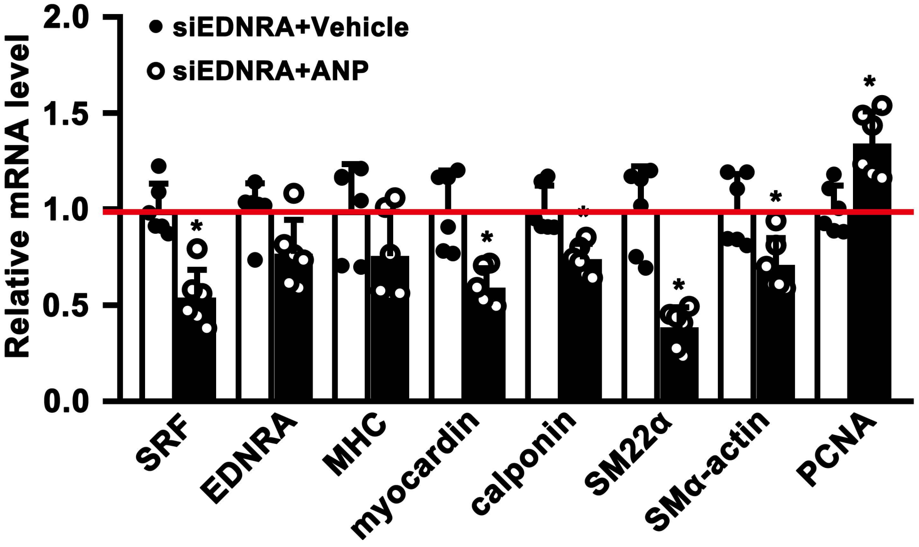

Relative mRNA level
2.0
1.5
1.0
0.5
0.0
siEDNRA+Vehicle
siEDNRA+ANP
SRF
EDNRA
MHC
myocardin
calponin
SM22α
SMα-actin
PCNA

S Figure 14

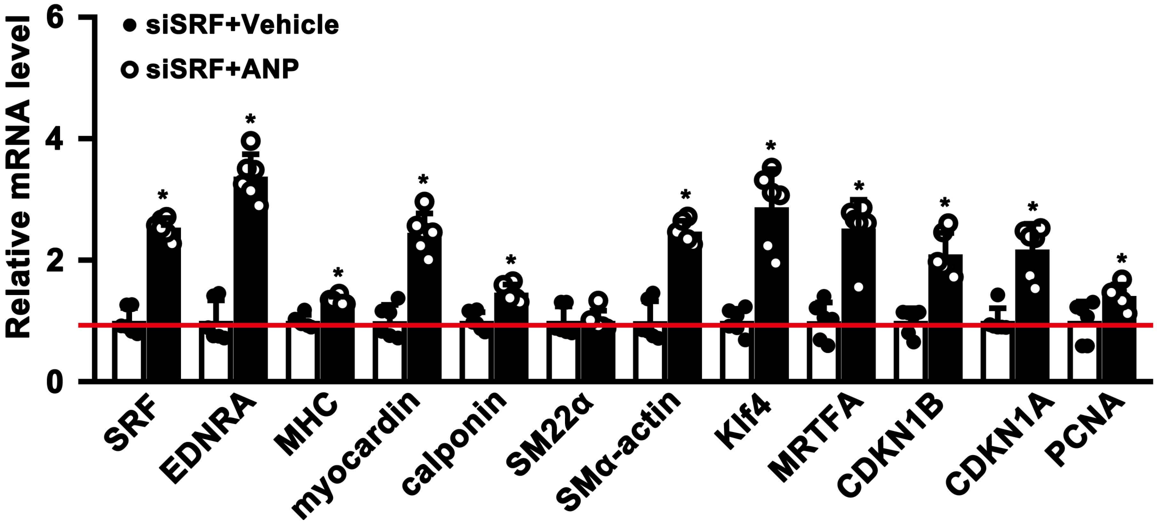

Relative mRNA level
6
4
2
0
siSRF+Vehicle
siSRF+ANP
SRF
EDNRA
MHC
myocardin
calponin
SM22α
SMα-actin
Klf4
MRTFA
CDKN1B
CDKN1A
PCNA

S Figure 15

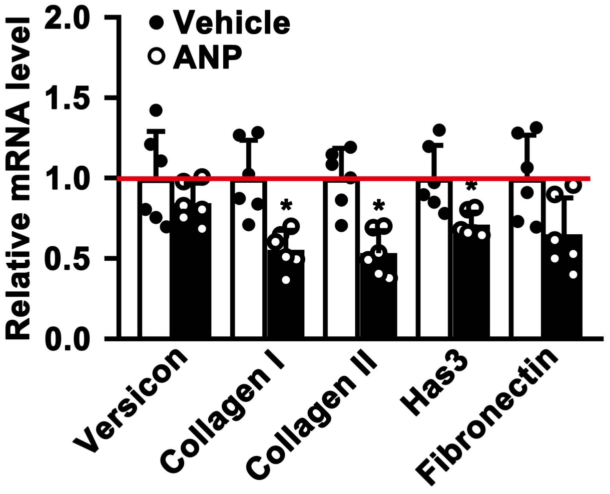

Relative mRNA level
2.0
1.5
1.0
0.5
0.0
Vehicle
ANP
*
*
*
Versicon
Collagen I
Collagen II
Has3
Fibronectin
